# In situ expansion and reprogramming of Kupffer cells elicit potent tumoricidal immunity against liver metastasis

**DOI:** 10.1172/JCI157937

**Published:** 2023-04-17

**Authors:** Wei Liu, Xia Zhou, Qi Yao, Chen Chen, Qing Zhang, Keshuo Ding, Lu Li, Zhutian Zeng

**Affiliations:** 1 Department of Oncology, The First Affiliated Hospital of the University of Science and Technology of China (USTC), Division of Life Sciences and Medicine, and; 2 The CAS Key Laboratory of Innate Immunity and Chronic Disease, School of Basic Medical Sciences, USTC, Hefei, Anhui, China.; 3 Department of Pathology, School of Basic Medicine, Anhui Medical University, Hefei, Anhui, China.; 4 Department of Pathology, The First Affiliated Hospital of Anhui Medical University, Hefei, Anhui, China.

**Keywords:** Hepatology, Immunology, Immunotherapy, Liver cancer, Macrophages

## Abstract

Liver metastasis represents one of the most frequent malignant diseases with no effective treatment. Functional reprogramming of Kupffer cells (KCs), the largest population of hepatic macrophages, holds promise for treating liver cancer, but remains seldom exploited. Taking advantage of the superior capacity of KCs to capture circulating bacteria, we report that a single administration of attenuated *Escherichia coli* producing clustered regularly interspersed short palindromic repeats CasΦ (CRISPR/CasΦ) machinery enables efficient editing of genes of interest in KCs. Using intravital microscopy, we observed a failure of tumor control by KCs at the late stage of liver metastasis due to KC loss preferentially in the tumor core and periphery, resulting in inaccessibility of these highly phagocytic macrophages to cancer cells. Simultaneous disruption of MafB and c-Maf expression using the aforementioned engineered bacteria could overcome KC dysfunction and elicit remarkable curative effects against several types of metastatic liver cancer in mice. Mechanistically, bacterial treatment induced massive proliferation and functional reprogramming of KCs. These cells infiltrated into the tumor, dismantled macrometastases by nibbling cancer cells, and skewed toward proinflammatory macrophages to unleash antitumor T cell responses. These findings provide an immunotherapy strategy that could be applicable for treating liver metastasis and highlight the therapeutic potential of targeting tissue-resident macrophages in cancer.

## Graphical abstract

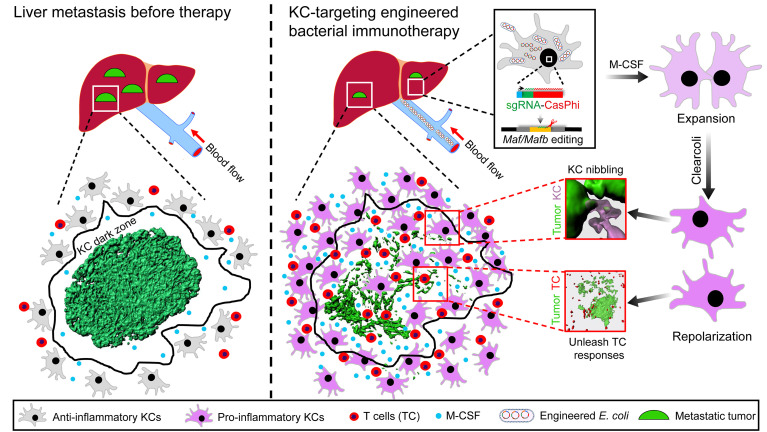

## Introduction

Hepatic malignancies account for a large number of cancer-related deaths. Although the incidence of primary liver cancer is on the rise due to the prevalence of chronic liver diseases, secondary liver cancer, i.e., liver metastasis, occurs 18 to 40 times more often than primary hepatocarcinogenesis (1). The unique anatomical microenvironment of the liver predisposes this organ to be a major metastatic site for extrahepatic cancer cells, including colorectal, pancreatic, breast, melanoma, and lung cancers. Liver metastasis is associated with poor prognosis in these cancer patients, and the majority of liver metastases are nonresectable with no current curative treatment (2, 3). Recent findings reported that liver metastasis could also lead to resistance to T cell–based cancer immunotherapy by inducing regulatory T cells and eliminating antigen-specific effector T cells (4, 5). Hence, there is an emergent need to develop new immunotherapy approaches against metastatic liver cancers.

Therapeutically targeting macrophages is promising for cancer immunotherapy. Hepatic macrophages are a remarkably heterogeneous population consisting of cells with distinct origins, locations, and functions in liver diseases (6, 7). While much attention has been focused on modulating bone marrow–derived infiltrating macrophages, functional reprogramming of Kupffer cells (KCs), which dominate the homeostatic tissue-resident macrophage pool in the body, has seldom been exploited in treating liver cancer (8). This is at least in part due to the lack of a convenient and efficacious method for in situ modification of these cells. KCs are developed from yolk-sac progenitors and are able to self-renew throughout life with minimal contribution from adult hematopoiesis (9, 10). They reside in the liver sinusoids and the space of Disse, forming a pivotal intravascular immune barrier that constantly filters the blood by rapidly recognizing, sequestering, and clearing circulating pathogens, foreign particles, cell debris, and other harmful substances (11). This high scavenging and phagocytic capacity of KCs makes them a central part of hepatic immunosurveillance against blood-borne metastases (12). KCs can directly uptake and clear circulating cancer cells via C-type lectins and Fc receptors, by which KCs suppress tumor seeding in the liver (13, 14). However, KCs were also reported to be protumoral at the late stage of liver metastasis (15). How this functional transition of KCs occurs during cancer progression remains elusive and will provide important implications for therapeutic intervention.

Increasing efforts have been made to engineer bacteria as therapeutic delivery vehicles because of their apparent advantages in terms of tumor tropism, low cost, easy handling, and potential for large-scale production (16). Recent advances in synthetic biology and cancer immunotherapy have greatly spurred the development of bacterial tumor therapy. Compelling therapeutic effects have been achieved in some preclinical studies utilizing bacteria to perform spatiotemporally controlled delivery of anticancer agents (17, 18). For example, engineered bacteria containing nanoantibodies, siRNAs, or immune stimulatory metabolites can preferentially accumulate in tumor tissues, release their therapeutic payloads into the tumor microenvironment, and lead to tumor regression (19–22). Whether the bacterial-based delivery system can be explored to target a specific immune cell type for the purpose of functionally modulating these cells in situ has not been tested thus far.

We have previously shown that circulating bacteria were rapidly and selectively captured by KCs (23, 24), which prompted us to speculate that bacteria may be used as plasmid DNA delivery vehicles enabling genetic modification and functional reprogramming of KCs in vivo. In this study, we tested this hypothesis and reported that a single administration of engineered *Escherichia coli* producing clustered regularly interspersed short palindromic repeats (CRISPR) machinery and attenuated LPS resulted in the efficient deletion of the gene of interest in KCs in vivo without inducing robust inflammation or compromising the integrity of the resident KC pool. Disruption of the transcription factors MafB and c-Maf in KCs by this approach can overcome tumor-induced KC loss and dysfunction and elicit unprecedented therapeutic effects against various types of metastatic liver cancer.

## Results

### Bacteria can be exploited as KC-targeting gene-delivery vehicles.

**Figure 1:**
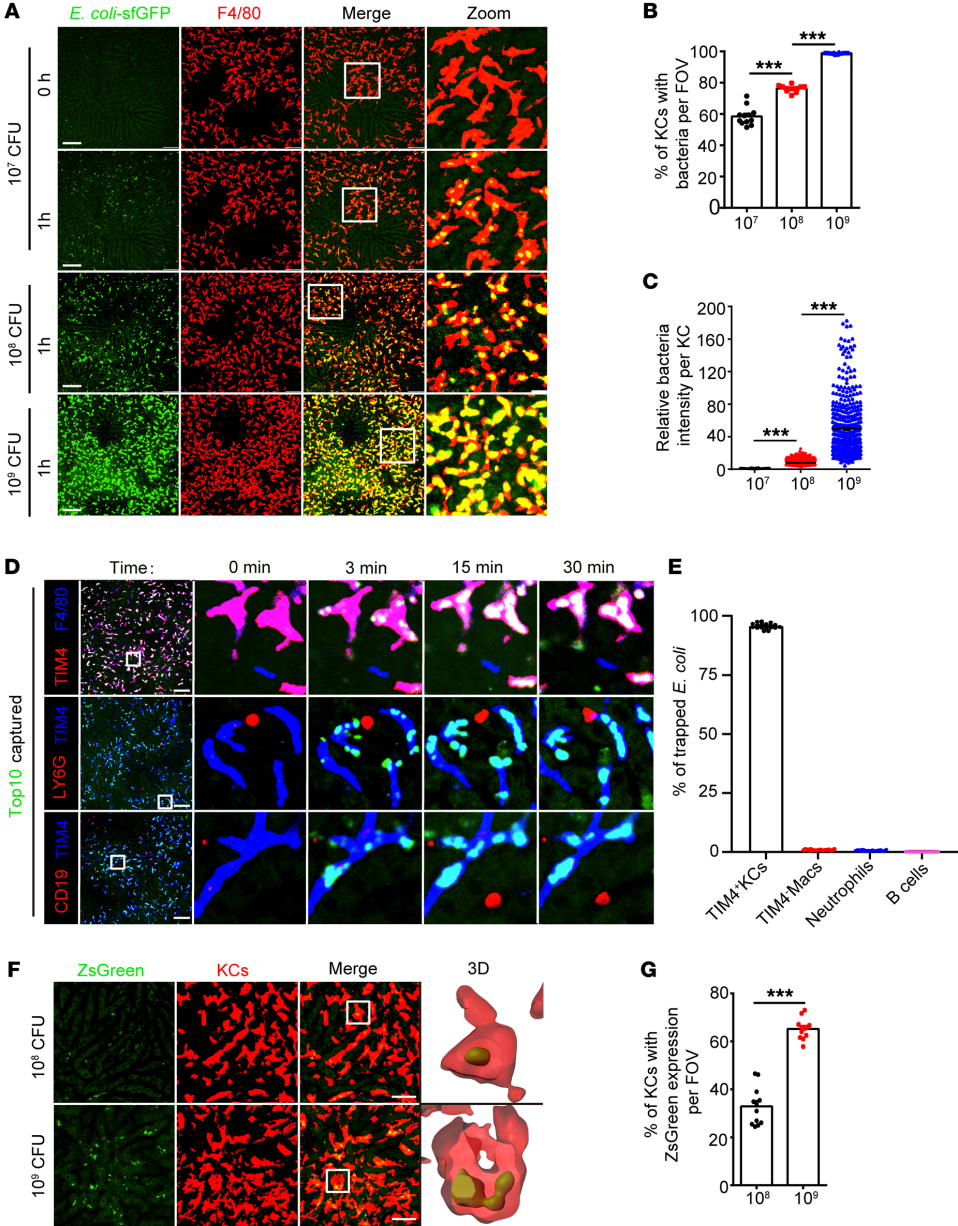
Exploiting bacteria as a KC-targeting plasmid delivery system. (**A**) Representative intravital images showing hepatic sequestration of sfGFP-tagged *E*. *coli* TOP10 at 1 hour after infection with the indicated doses of bacteria. Scale bars: 100 μm. Original magnification, zoomed images: ×3.6. (**B**) The percentages of KCs that captured *E*. *coli* per field of view (FOV) were quantified. (**C**) The normalized sfGFP fluorescence intensity of bacteria-containing KCs. Each circle represents 1 KC. (**D**) Representative intravital images and (**E**) statistics for bacterial capture by TIM4^+^ resident KCs, TIM4^–^ macrophages, neutrophils, or B cells. Scale bars: 100 μm. Original magnification, zoomed images: ×9.0. (**F**) Representative liver images at 24 hours after infection with the indicated doses of *E*. *coli* TOP10 harboring mammalian expression plasmids for ZsGreen. Scale bars: 40 μm. Original magnification, zoomed images: ×5.6. (**G**) Percentages of TIM4^+^ resident KCs with ZsGreen expression per FOV. For **B**, **D** and **F**, each circle represents 1 FOV, and a total of 12 to 20 randomly selected FOVs from 3 mice are shown. Representative data from 2 independent experiments are shown. Data are represented as mean ± SEM. ***P < 0.001, 1-way ANOVA with Tukey’s test (**B** and **C**); unpaired Student’s *t* test (**G**).

A prerequisite of using bacteria as KC-targeting delivery vehicles is to ensure that all KCs take up bacteria. To this end, we studied the ability of KCs to capture circulating bacteria at various infectious doses using intravital microscopy (IVM). In line with our previous findings (23, 24), i.v. injected *E*. *coli* TOP10 expressing superfolder GFP (sfGFP) was arrested almost exclusively by F4/80^+^TIM4^+^ KCs, but not neutrophils, B cells, and F4/80^+^TIM4^–^ macrophages in the liver regardless of the size of the inoculum (Figure 1, A–E, Supplemental Figure 1, A and B, and Supplemental Video 1; supplemental material available online with this article; https://doi.org/10.1172/JCI157937DS1). The percentage of KCs taking up bacteria increased from 60% to 80% and 99% at infectious doses of 10^7^, 10^8^, and 10^9^ CFU *E*. *coli*, respectively. At the highest dose, each KC engulfed a large number of bacteria, as reflected by a strong intracellular GFP signal that nearly filled the whole cell body, making it impossible to distinguish individual bacterium (Figure 1, A–C). It was worth noting that this high-dose infection did not alter the tissue-distribution pattern of *E*. *coli*. The liver remained a primary site for sequestering circulating bacteria, showing at least 10-fold more *E*. *coli* in this organ than any other tested tissues, and the vast majority of them were cleared within a week (Supplemental Figure 1, C–E). We surmised that a high intracellular bacterial load may increase the chance of *E*. *coli* escape from phagosomes into the cytosol, by which KCs can be transfected by bacteria-derived plasmid DNA. To test this hypothesis, we injected mice with *E*. *coli* harboring a plasmid that directs ZsGreen synthesis in mammalian but not bacterial cells. Interestingly, approximately 70% of KCs, but not any other hepatic cell types, exhibited strong ZsGreen fluorescence after bacterial injection at a dose of 10^9^ CFU, indicating the successful delivery of plasmid DNA specifically to KCs. Lowering the bacterial inoculum to 10^8^ CFU dramatically impaired plasmid delivery in KCs. Only 30% of KCs showed ZsGreen expression, and its fluorescence intensity was much lower on a per cell basis than that of a high-dose infection (Figure 1, F and G, and Supplemental Figure 1, F–I). These data thus demonstrated the feasibility of utilizing high-dose bacterial injection as a method for KC-specific gene delivery.

### Genetic modification of KCs in situ via bacterial delivery of CRISPR machinery.

**Figure 2:**
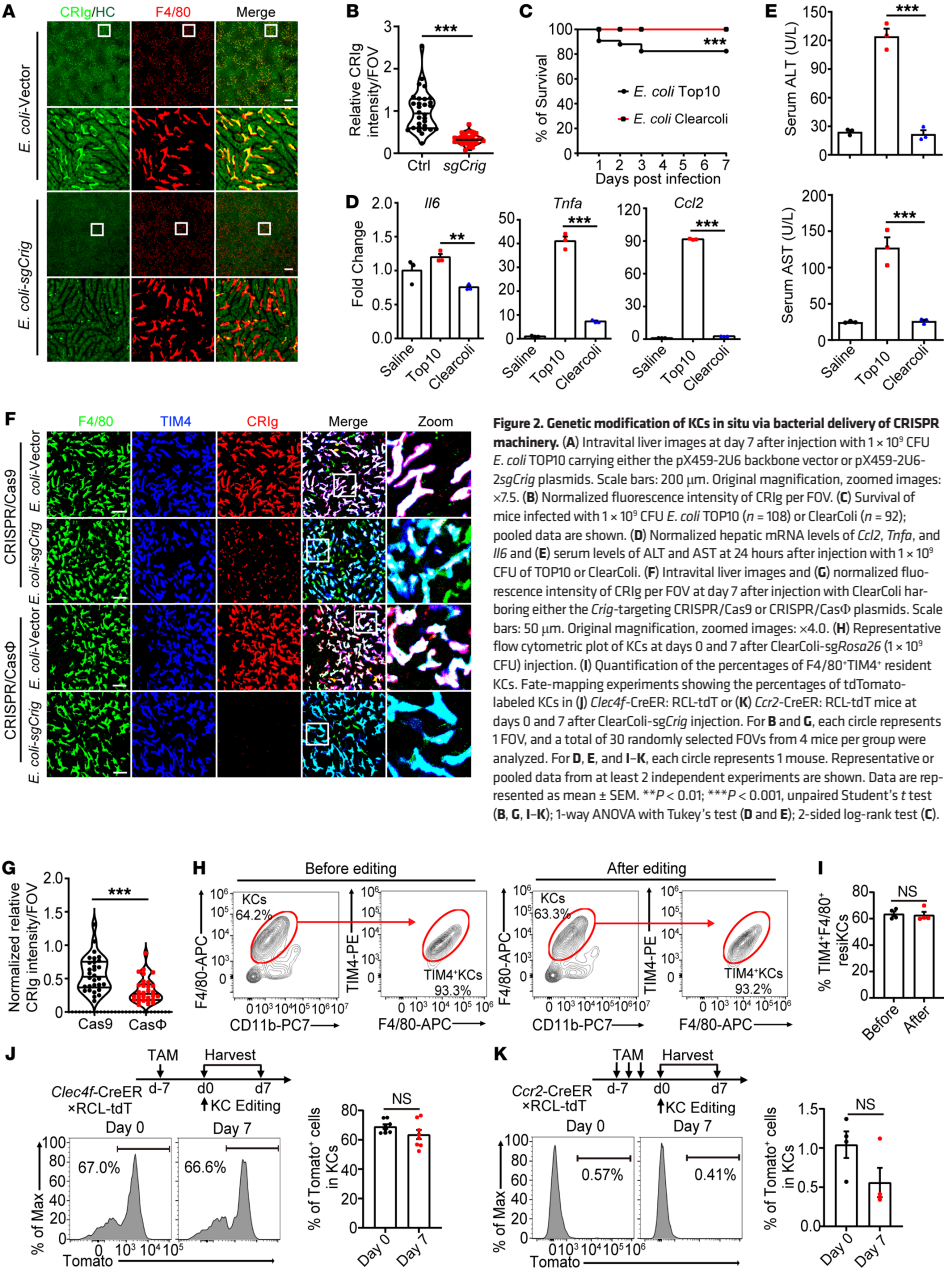
Genetic modification of KCs in situ via bacterial delivery of CRISPR machinery. (**A**) Intravital liver images at day 7 after injection with 1 × 10^9^ CFU *E*. *coli* TOP10 carrying either the pX459-2U6 backbone vector or pX459-2U6-2*sgCrig* plasmids. Scale bars: 200 μm. Original magnification, zoomed images: ×7.5. (**B**) Normalized fluorescence intensity of CRIg per FOV. (**C**) Survival of mice infected with 1 × 10^9^ CFU *E*. *coli* TOP10 (*n* = 108) or ClearColi (*n* = 92); pooled data are shown. (**D**) Normalized hepatic mRNA levels of *Ccl2*, *Tnfa*, and *Il6* and (**E**) serum levels of ALT and AST at 24 hours after injection with 1 × 10^9^ CFU of TOP10 or ClearColi. (**F**) Intravital liver images and (**G**) normalized fluorescence intensity of CRIg per FOV at day 7 after injection with ClearColi harboring either the *Crig*-targeting CRISPR/Cas9 or CRISPR/CasΦ plasmids. Scale bars: 50 μm. Original magnification, zoomed images: ×4.0. (**H**) Representative flow cytometric plot of KCs at days 0 and 7 after ClearColi-sg*Rosa26* (1 × 10^9^ CFU) injection. (**I**) Quantification of the percentages of F4/80^+^TIM4^+^ resident KCs. Fate-mapping experiments showing the percentages of tdTomato-labeled KCs in (**J**) *Clec4f*-CreER: RCL-tdT or (**K**) *Ccr2*-CreER: RCL-tdT mice at days 0 and 7 after ClearColi-sg*Crig* injection. For **B** and **G**, each circle represents 1 FOV, and a total of 30 randomly selected FOVs from 4 mice per group were analyzed. For **D**, **E**, and **I**–**K**, each circle represents 1 mouse. Representative or pooled data from at least 2 independent experiments are shown. Data are represented as mean ± SEM. **P < 0.01; ***P < 0.001, unpaired Student’s *t* test (**B**, **G**, **I**–**K**); 1-way ANOVA with Tukey’s test (**D** and **E**); 2-sided log-rank test (**C**).

Cellular delivery of the CRISPR/Cas machinery is widely used for genome editing in vivo and holds great therapeutic promise. To determine whether we could edit the gene of interest in KCs using bacterial delivery of CRISPR/Cas9 plasmids, we chose CRIg, F4/80, and TIM4 as readouts because they were highly expressed on the surface of KCs and can be readily detectable by IVM at the single-cell level. Simultaneous use of dual sgRNAs targeting different exons of a given gene can significantly enhance the efficacy of CRISPR-mediated gene deletion (25). For this purpose, we duplicated the sgRNA expression cassette of the commonly used CRISPR/Cas9 vector *pX459* (26) and constructed it to encode 2 individual *Crig*-targeting sgRNAs (*pX459-2U6-2sgCrig*; Supplemental Figure 2, A and B). WT mice injected with 10^9^ CFU of *E*. *coli* TOP10 harboring this plasmid displayed a dramatic reduction in CRIg expression in the liver, with the majority of KCs showing complete absence of CRIg (Figure 2, A and B, and Supplemental Figure 2, C and D). In contrast, mice injected with *E*. *coli* containing the *pX459-2U6* backbone vector retained high levels of CRIg expression, excluding the possibility of bacteria-induced inflammation in downregulating CRIg expression. Similarly, i.v. injection of *E*. *coli* containing *Timd4-* or *Adgre1*-targeting plasmids decreased the levels of TIM4 or F4/80 on KCs without affecting CRIg expression (Supplemental Figure 2, E–G), corroborating a specific editing of the gene of interest in KCs by this method, although the efficiency varies among different genes. To edit multiple genes in KCs simultaneously, we adopted an approach cotransfecting the *Crig-* and *Adgre1*-targeting CRISPR/Cas9 plasmids into *E*. *coli* via electroporation, enabling bacterial cells to carry high copies of both plasmids. Injection of these bacteria led to drastically decreased expression of both CRIg and F4/80 in anti-TIM4–labeled KCs (Supplemental Figure 2, H and I). Taken together, these data suggest that using bacteria as CRISPR/Cas9 plasmid delivery vehicles enabled in situ genetic modification of KCs.

Although the *E*. *coli* TOP10 we used above was a nonpathogenic strain, it can still cause endotoxemia in a proportion of mice, leading to a 20% mortality rate when injected at a high dose (Figure 2C). This was accompanied by robust inflammatory responses in the liver (Figure 2, D and E, and Supplemental Figure 3, A and B), resulting in partial loss of TIM4^+^ resident KCs and their replenishment by monocyte-derived TIM4^–^ KCs (Supplemental Figure 3, C and D), which would compromise the feasibility of using TOP10 as KC-targeting gene-delivery vehicles. We therefore investigated whether these inflammatory responses could be prevented by using ClearColi, which is an electrocompetent *E*. *coli* strain with genetically attenuated LPS. This modified LPS was devoid of the O-side chain and had altered lipid A so that these bacteria were disabled from mounting LPS-related immune responses (27). Indeed, mice injected with 10^9^ CFU of ClearColi all survived without showing significant signs of sepsis (Figure 2C). These bacteria were still rapidly sequestered in the liver mainly by resident KCs, but not neutrophils, B cells, and F4/80^+^TIM4^–^ macrophages (Supplemental Figure 4, A–D, and Supplemental Video 2), yet the hepatic expression of canonical proinflammatory cytokines, including IL-6, TNF-α, and C-C motif chemokine ligand 2 (CCL2), was either not changed or only slightly elevated compared with that in control mice with saline injection (Figure 2D). Consistently, ClearColi mobilized only mild and transient infiltration of neutrophils (Supplemental Figure 3, A and B). As a consequence, no collateral liver damage was induced, as evaluated by serum levels of alanine aminotransferase (ALT) and aspartate aminotransferase (AST) (Figure 2E). Importantly, the TIM4^+^ resident KC pool remained intact upon high-dose ClearColi injection, without showing a striking reduction in these cells or their replenishment by TIM4^–^ monocyte–derived KCs (Supplemental Figure 3, C and D). It is worth mentioning that ClearColi-induced inflammatory responses could be even milder in humans than what we observed in mice because the altered lipid A of ClearColi (lipid Iva) can still act agonistically to mouse but not human TLR4/MD2 (28). Therefore, using LPS-attenuated *E*. *coli* bacteria for KC-targeting delivery of CRISPR/Cas plasmids can greatly improve the safety of our method by relieving endotoxin-induced overwhelming inflammation.

However, we observed suboptimal gene editing of KC by ClearColi compared with the TOP10 strain (Supplemental Figure 5A). This was possibly because ClearColi produced fewer plasmids in culture than TOP10 did, resulting in insufficient plasmid delivery to KCs (Supplemental Figure 5B). CRISPR/CasΦ was recently reported as a hypercompact genome editor with apparent advantages in vector-based cellular delivery (29). To determine whether this system can be adopted to improve the efficacy of ClearColi-mediated gene editing in KCs, we constructed a dual-sgRNA CRISPR/CasΦ vector (29) encoding CasΦ-2 nuclease and 2 *Crig*-targeting sgRNAs (*pPP441-2U6-2sgCrig*) (Supplemental Figure 5C). As compared with the *pX459-2U6-2sgCrig* CRISPR/Cas9 plasmid, mice injected with ClearColi harboring this CRISPR/CasΦ plasmid displayed a more profound reduction in CRIg expression (Figure 2, F and G). Kinetical analysis showed that CRIg expression declined as early as 2 days after bacterial injection, reached a maximum reduction at day 7, and was maintained at this low level for at least 45 days (Supplemental Figure 5, D and E). These data collectively proved the superiority of using CRISPR/CasΦ for highly efficient and long-lasting in situ gene editing of KCs.

We next assessed the inflammatory responses and tissue toxicity elicited by ClearColi-CRISPR/CasΦ system–mediated gene editing in KCs. Here, we chose to target the Rosa 26 locus instead of CRIg, TIM4, or F4/80 to avoid the potential immune-modulatory function of these genes. As expected, no elevation of serum levels of ALT or AST was observed at all time points examined after injection of *Rosa26*-targeting ClearColi (Supplemental Figure 6A). Intrahepatic expression of proinflammatory cytokines (*Il6*, *Il1b*, *Tnfa*, *Cxcl2*, *Cxcl1*, and *Ccl2*) was either not changed or only moderately and transiently upregulated (Supplemental Figure 6B), in accordance with a mild and transient infiltration of neutrophils (Supplemental Figure 6, C and D). Most importantly, ClearColi-mediated gene editing did not result in a reduction in F4/80^+^TIM4^+^-resident KCs (Figure 2, H and I, and Supplemental Figure 6E). To further confirm this, we leveraged the fate-mapping strategy and generated a *Clec4f*-CreER×R26-LSL-tdTomato mouse strain, in which tamoxifen-induced Cre expression in KCs can cleave the STOP codon and enable permanent labeling of these cells. We showed that a single administration of tamoxifen was sufficient to specifically label two-thirds of KCs, validating the feasibility of this method (Supplemental Figure 6F). The proportion of tdTomato-labeled KCs remained unchanged after ClearColi-mediated gene editing (Figure 2J), strongly indicating the intactness of the resident KC pool. We also used *Ccr2*-CreER×R26-LSL-tdTomato mice to trace monocytes and their derivatives. While blood monocytes were efficiently labeled (Supplemental Figure 6G), less than 1% of KCs were tdTomato^+^ after recombinant ClearColi injection, suggesting no replacement of KCs by monocyte-derived cells (Figure 2K). Altogether, we have established an optimized method for efficient disruption of the gene of interest in KCs using engineered bacteria without inducing significant inflammation or compromising the integrity of the resident KC pool. We termed this method bacterial-mediated in situ gene editing of liver-resident macrophages by CRISPR (BIL-CRISPR).

### Impaired control of liver metastasis was associated with KC loss preferentially in the tumor core and periphery.

Having established a method for genetic modification of KCs in situ, we tested to determine whether this could be used to functionally manipulate KCs in diseases. Indeed, KCs of *Crig*-edited mice showed a diminished capacity to capture *Staphylococcus aureus* during bloodstream infections (Supplemental Figure 7), phenocopying CRIg knockout mice (23). In addition, BIL-CRISPR–mediated editing of *Trem1* — a well-characterized amplifier of inflammation expressed on KCs (30) — almost completely abolished concanavalin-induced (ConA-induced) acute hepatitis and prevented the rapid loss of resident KCs in ConA-treated mice (Supplemental Figure 8). These data highlighted the therapeutic potential of using BIL-CRISPR to modulate the function and fate of KCs in liver diseases.

**Figure 3:**
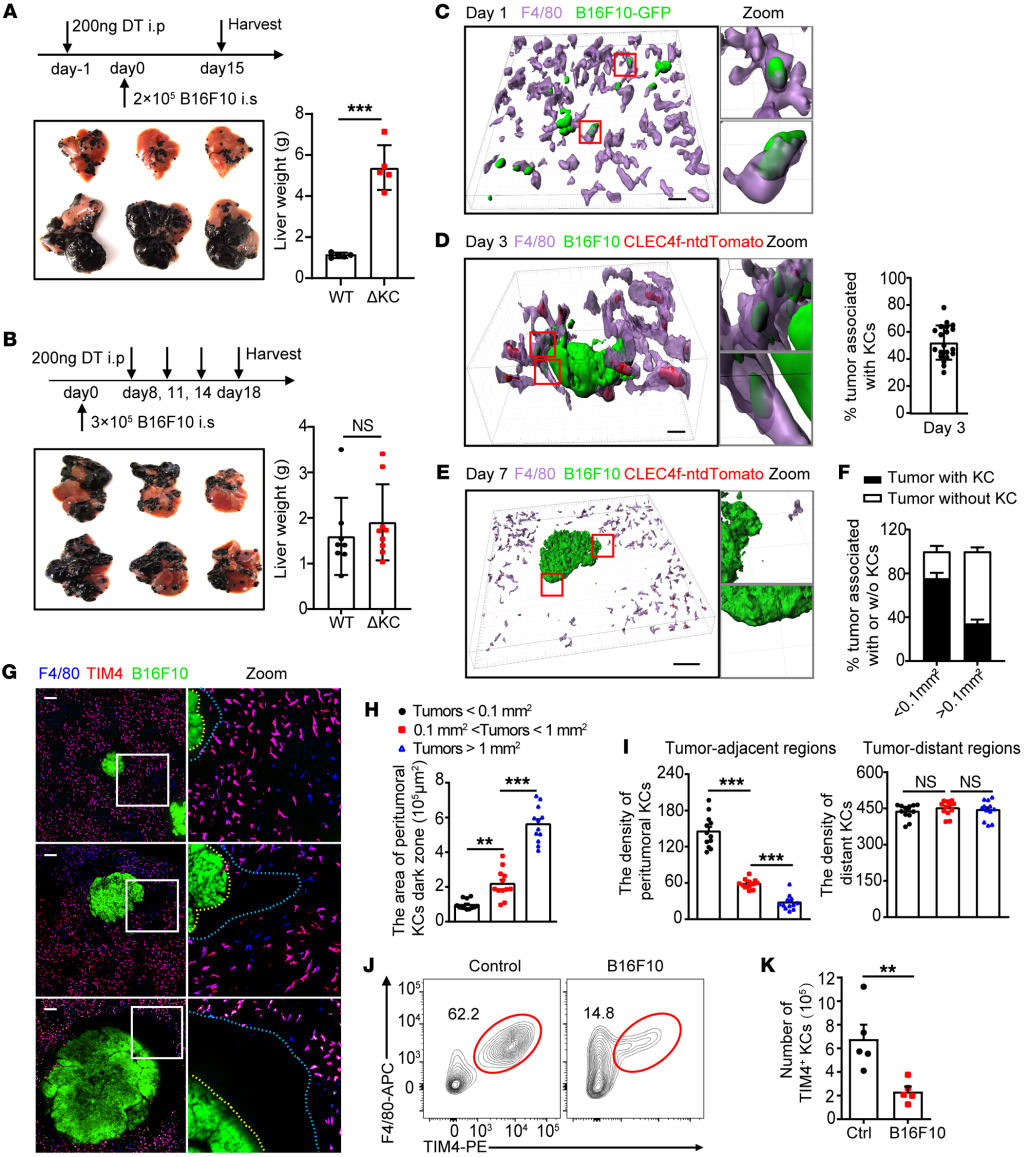
Loss of KCs preferentially in the tumor core and periphery at the late stage of liver metastasis. (**A**) *Clec4f*-iDTR and WT C57BL/6 mice were treated with DT prior to or (**B**) after B16F10 tumor cell inoculation, and their livers were harvested at the indicated time points. *n* = 5 mice per group. Mice with intrasplenically injected B16F10-ZsGreen cells were imaged at (**C**) day 1, (**D**) day 3, or (**E**) day 7 after injection. Scale bars: 40 (**C**); 20 (**D**); 100 μm (**E**). Original magnification, zoomed images: ×4.3 (**C**); ×3.8 (**D**); ×4.3 (**E**). Tumors that closely interacted with at least 3 KCs were considered KC-associated tumors, and their ratio was quantified at day 3 (**D**: right panel) and (**F**) day 7. (**G**) Localization of KCs with tumors of different sizes. KC dark zones are outlined between dashed lines. Scale bars: 140 μm. Original magnification, zoomed images: ×3.0. (**H**) Quantification of the area of the KC dark zone in **G**. (**I**) Quantification of the density of KCs in tumor-adjacent (0–200 μm away from the tumor edge) or distant areas (600–800 μm away from the tumor edge). For **H** and **I**, each circle represents 1 tumor. *n* = 12. (**J**) Representative flow cytometric plot of F4/80^+^TIM4^+^ resident KCs in tumor-free or tumor-bearing mice. (**K**) KC numbers per mouse are shown. *n* = 5 mice. Representative or pooled data from at least 2 independent experiments are shown. Data are represented as mean ± SEM. **P < 0.01; ***P < 0.001, unpaired Student’s *t* test (**A**, **B** and **K**); 1-way ANOVA with Tukey’s test in (**H** and **I**).

Bacteria-mediated tumor therapy represents a promising therapeutic alternative for cancers. To exploit the application of BIL-CRISPR in treating metastatic liver cancer, we set out to examine the function of KCs during disease progression using *Clec4f*-iDTR mice, in which KCs can be specifically depleted upon diphtheria toxin (DT) administration without affecting monocytes and other tissue-resident macrophages (31). In a well-established intrasplenic injection model of liver metastasis, KC depletion prior to tumor cell injection induced outgrowth of liver metastasis, confirming a critical role of KCs in restricting hepatic tumor development (Figure 3A). However, depleting KCs after the onset of liver macrometastasis had no effect on tumor growth, indicating that the aforementioned antitumor function of KC was suppressed in established metastatic liver cancer (Figure 3B). We studied the underlying mechanisms by delineating the dynamic interactions between KCs and tumors during disease progression. Intravital imaging revealed that KCs rapidly arrested a proportion of metastatic B16F10-GFP melanoma cells that entered the liver sinusoids. Although these arrested tumor cells were not entirely engulfed (Supplemental Figure 9A), they seemed to be gradually ripped off by neighboring KCs (Supplemental Figure 9B and Supplemental Video 3). In line with this, many KCs internalized GFP^+^ tumor–derived particles at day 1 after tumor inoculation (Figure 3C). At day 3, when small metastatic colonies appeared, nearly 50% of these micrometastases were encased by KCs, which intimately interacted with tumors and ingested tumor fragments (Figure 3D). Some KCs can even penetrate inside the tumor core, as shown in *Clec4f-*tdTomato mice, which express nuclear-localized tdTomato in KCs (32), leading to tumor dissociation (Supplemental Figure 9C). It was therefore speculated that physically interacting with tumor cells was fundamental to KC-mediated early control of liver metastasis.

However, at day 7, when large metastatic tumors (macrometastasis) developed, tdTomato^+^ KCs were rarely detected in the core and periphery of these large tumors (Figure 3E). The scarcity of KCs in the tumor periphery was further confirmed using antibody labeling of F4/80, TIM4, and CRIg, clearly showing a “dark zone” surrounding hepatic macrometastases (Figure 3, E and F, and Supplemental Figure 10A). This peritumoral KC dark zone became even larger in size with tumor progression, forming a barrier that impeded the access of these phagocytes to cancer (Figure 3, G and H). Interestingly, the peritumoral areas remained well perfused by TRITC-Dextran, indicating an intact sinusoidal structure and excluding the possibility of antibody impenetrability in these areas during in vivo labeling of KCs (Supplemental Figure 10B). We then postulated that the appearance of the KC dark zone was a result of tumor-induced KC loss. In fact, the density of KCs was lower in tumor-adjacent versus tumor-distant tissues in the liver (Figure 3I). Tumor-induced loss of F4/80^+^TIM4^+^ tissue-resident KCs was further confirmed using flow cytometry, showing an overall 3-fold decrease in these cells in tumor-bearing livers (Figure 3, J and K). We tried to characterize the death pathway responsible for KC loss. Although peritumoral KCs were rarely labeled with propidium iodide (PI) (Supplemental Figure 10C), tumor-enriched liver tissues had a higher proportion of annexin V^+^KCs than tumor-scarce liver tissues (Supplemental Figure 10, D–F), implying that apoptosis rather than necroptosis could play at least a partial role in tumor-induced KC loss. Taking these data together, KCs perform a critical immune-surveillance function by directly ingesting cancer cells at the early stage of liver metastasis. This antitumor function was impaired thereafter, at least partially due to the preferential loss of KCs in the peritumoral region, leading to inaccessibility of these highly phagocytic macrophages to cancer cells. Similar findings were also observed in liver samples from patients with colorectal liver metastasis. Using MARCO as a discriminative marker for resident KCs in humans (33), we found that KCs were enriched in tumor-distant liver tissues, but were scarce in the tumor core and periphery (Supplemental Figure 11, A and B). We therefore hypothesized that increasing the abundance of intratumoral KCs may be beneficial for treating liver cancers. In support of this, intertumoral expression levels of KC signature genes, particularly *TIMD4* and *CLEC1B* (34), were positively associated with the survival rates of hepatocellular carcinoma patients (Supplemental Figure 11, C and D).

### Disruption of MafB/c-Maf by BIL-CRISPR exhibited striking prophylactic and therapeutic effects against liver metastasis.

**Figure 4:**
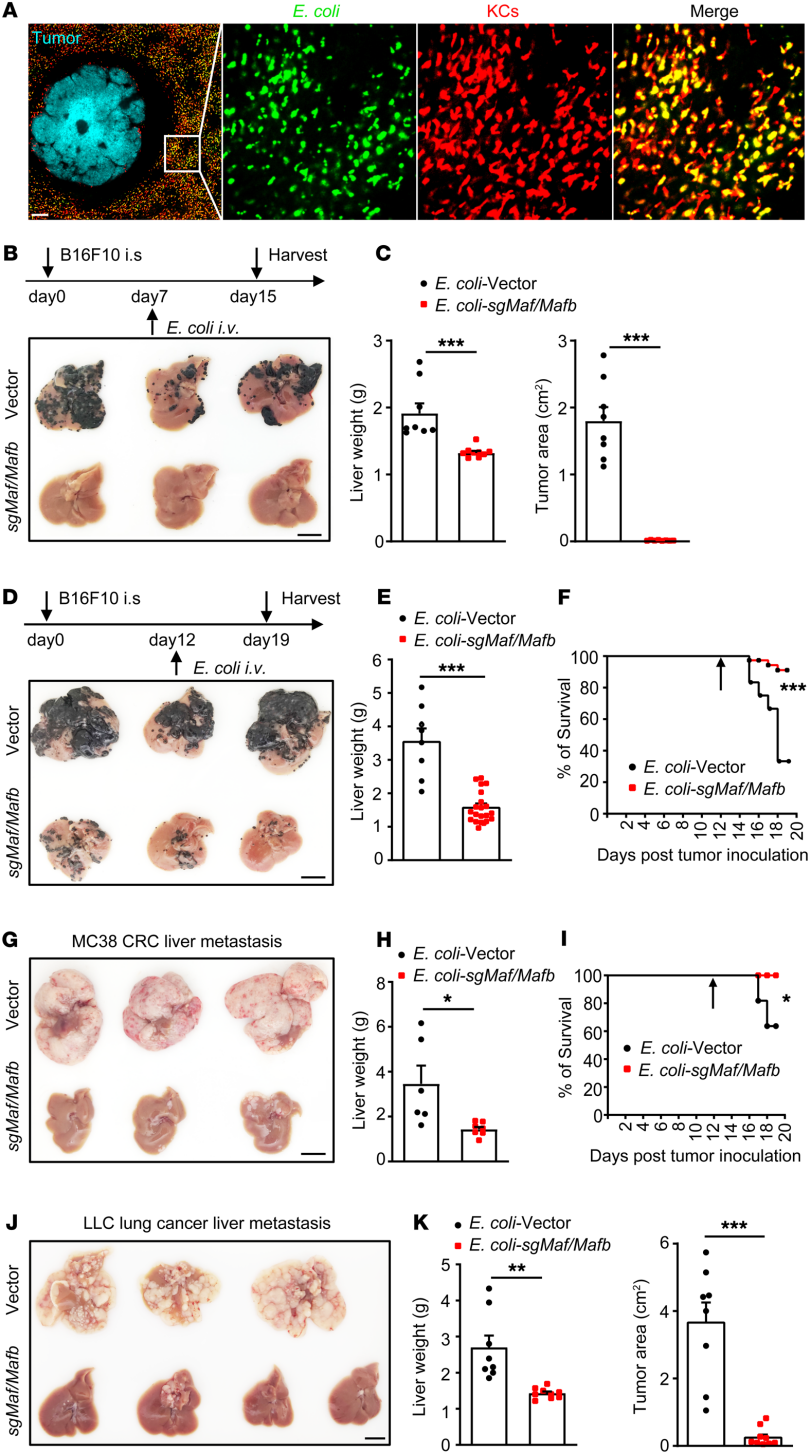
Therapeutic effects against liver metastasis by bacteria-mediated disruption of c-Maf/MafB in KCs. (**A**) Representative images showing ClearColi captured by KCs at 1 hour after infection in tumor-bearing mice. Scale bars: 200 μm. Original magnification, zoomed images: × 5.6. (**B**) Mice were treated with *E*. *coli*–vector or *E*. *coli*–*sgMafb/Maf* at day 7 after B16F10 tumor inoculation and were harvested at day 15 as illustrated. (**C**) Liver weight and tumor area on the surface of the liver were quantified. *n* = 8–9 mice per group pooled from 2 experiments. (**D**) Treatment of late-stage B16F10 melanoma liver metastasis as depicted. (**E**) Liver weights at day 19 were measured. (**F**) Mouse survival was monitored. Pooled data of 22–25 mice per group from 4 independent experiments. (**G**–**I**) Treatment of late-stage MC38 liver metastasis. Pooled data of 8–11 mice per group from 2 independent experiments. (**J**–**K**) Treatment of late-stage LLC liver metastasis. Pooled data of 8–9 mice per group from 2 independent experiments. Scale bars: 1 cm (**B**, **D**, **G**, and **J**). For **E** and **H**, mice that reached the end point of the experiment were euthanized before harvest and were excluded from liver weight analysis. Arrows in **F** and **I** indicate time points of bacterial treatment. Data are represented as mean ± SEM. *P < 0.05; **P < 0.01; ***P < 0.001, unpaired Student’s *t* test (**C**, **E**, **H** and **K**); 2-sided log-rank test (**F** and **I**).

Macrophages deficient in MafB and c-Maf (encoded by *Mafb* and *Maf*, respectively) can undergo robust proliferation without loss of differentiated phenotype and function (35, 36). We thus tested to determine whether BIL-CRISPR–mediated inactivation of these genes could be used to treat liver metastasis by expanding KCs in vivo. Given that the spleen was able to trap some of the injected bacteria but was removed in our mouse model of liver metastasis, we first demonstrated that splenectomy did not affect the gene-editing efficiency of BIL-CRISPR (Supplemental Figure 12, A and B). We then engineered ClearColi to carry both *Mafb-* and *Maf*-targeting dual sgRNA CRISPR-CasΦ plasmids (referred to as *E*. *coli*–sg*Mafb/Maf* hereinafter). Preinjecting these bacteria but not control bacteria (ClearColi containing backbone vector, hereinafter *E*. *coli*–vector) almost completely prevented metastatic melanoma growth in the liver (Supplemental Figure 12, C and D). Successful deletion of targeted DNA fragments of the *Mafb* and *Maf* genes was also validated in sorted KCs (Supplemental Figure 12, E and F). To explore the therapeutic potential of this approach against established liver metastasis, we first confirmed that the vast majority of i.v. injected *E*. *coli* were still rapidly captured by KCs, but not neutrophils, B cells, or TIM4^–^ macrophages and did not accumulate in tumors (Figure 4A and Supplemental Figure 4, E and F). After allowing liver macrometastasis to develop (7 days after tumor cell inoculation), mice were subjected to *E*. *coli*–vector or *E*. *coli*–sg*Mafb/Maf* injection. The latter treatment induced drastic tumor regression, showing an over 90% reduction in hepatic tumors at 7 days after bacteria injection compared with the *E*. *coli* vector (Figure 4, B and C). To mimic clinically relevant scenarios, we also treated mice at late time points after the induction of liver metastasis (i.e., at day 12 after tumor inoculation, since mice started reaching the end point of the experiment around day 15 in our model). Surprisingly, a single injection of *E*. *coli*–sg*Mafb/Maf* was sufficient for eradicating the majority of melanoma tumors in the liver and greatly improved the survival rate of tumor-bearing mice, from 30% to 90% (Figure 4, D–F), whereas disruption of either *Mafb* or *Maf* alone exhibited only a moderate reduction in hepatic tumor burden (Supplemental Figure 13, A and B). The profound therapeutic effect against late-stage metastatic liver cancer by *E*. *coli*–sg*Mafb/Maf* injection was also observed in MC38 colorectal cancer (CRC) and Lewis lung carcinoma (LLC) lung cancer liver metastases (Figure 4, G–K). Together, these results demonstrate that BIL-CRISPR–mediated simultaneous disruption of MafB and c-Maf in KCs provoked remarkable antitumor effects against metastatic liver cancers.

### KCs proliferated, infiltrated into tumors, and ingested cancer cells upon BIL-CRISPR–mediated inactivation of MafB/c-Maf.

**Figure 5:**
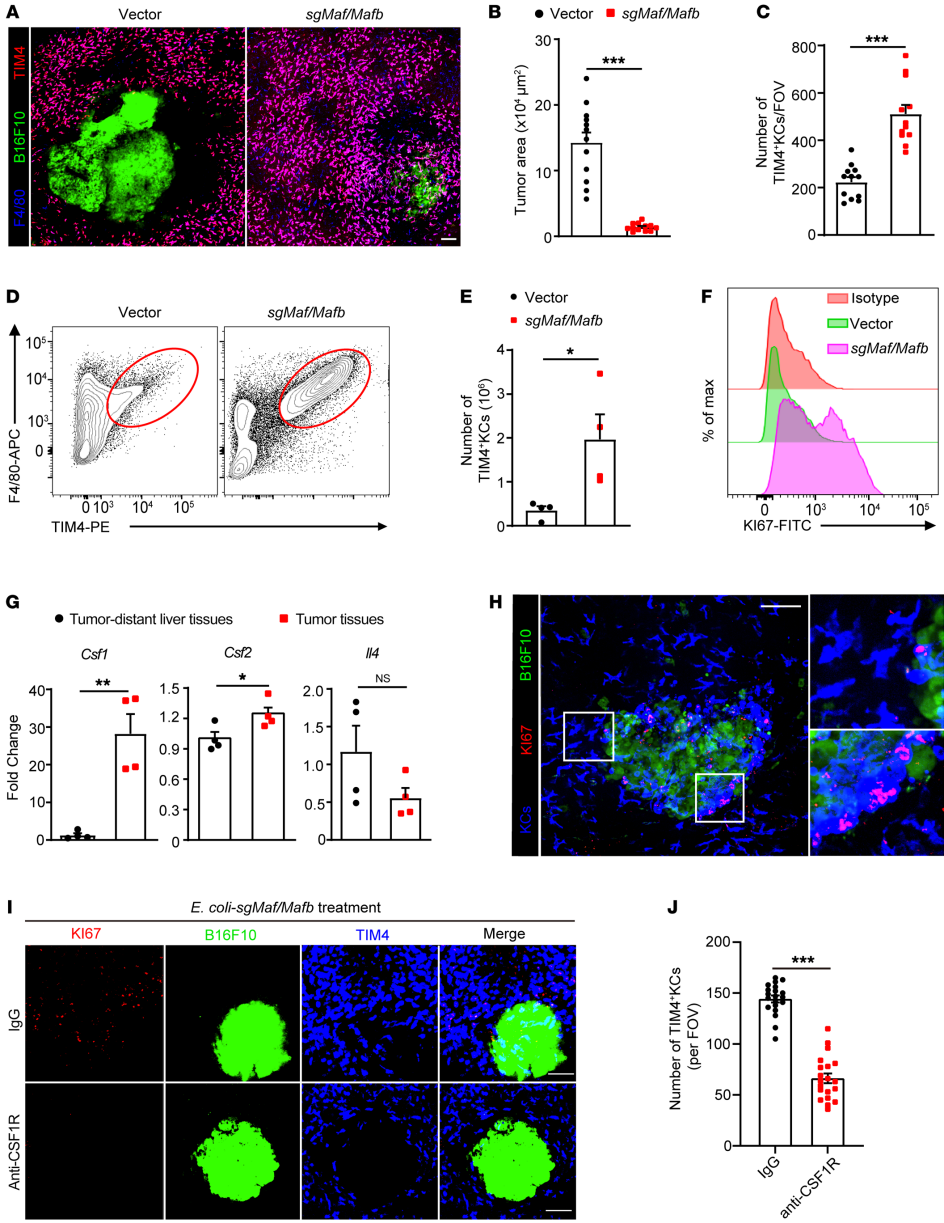
Massive proliferation of KCs during BIL-CRISPR–mediated bacterial therapy. (**A**) Intravital liver images of B16F10-ZsGreen tumor-bearing mice 7 days after bacterial treatment. Scale bar: 100 μm. (**B**) The area of ZsGreen^+^ tumors and (**C**) the number of KCs per FOV in **A** were quantified. A total of 12 randomly selected FOVs from 3 mice per group were analyzed. Mice at day 12 after intrasplenic B16 tumor injection were treated with *E*. *coli*–vector or *E*. *coli*–*sgMafB/Maf* and were harvested at day 19. (**D**) Representative flow cytometric plots of F4/80^+^TIM4^+^ tissue-resident KCs are shown. (**E**) Number of KCs in **D**. *n* = 4 mice per group. (**F**) Representative histogram of Ki67 expression in TIM4^+^F4/80^+^ KCs. (**G**) Normalized mRNA levels of *Csf1*, *Csf2*, and *Il4* in tumor tissues versus tumor-distant liver tissues in bacteria-treated tumor-bearing mice. *n* = 4 mice per group. (**H**) Ki67-RFP mice with B16F10-ZsGreen liver metastasis were treated with *E*. *coli*–*sgMafb/Maf* i.v. Representative intravital liver images and enlarged pictures are shown at 2 days after bacterial treatment. Original magnification, zoomed images: × 2.8. (**I**) Ki67-RFP mice were i.p. injected with control IgG or CSF1R antibody at days 6, 8, and 10 after inoculation of B16F10-ZsGreen tumors, bacterial therapy was performed at day 7, and liver images were taken 4 days after bacterial treatment. (**J**) The number of TIM4^+^ KCs per FOV in **I**. A total of 20 FOVs from 3 mice per group were analyzed. Representative data from 2 independent experiments are shown. Data are represented as mean ± SEM. *P < 0.05; **P < 0.01; ***P < 0.001, unpaired Student’s *t* test.

We next determined whether our bacterial therapy indeed induced KC expansion. Mice with established liver metastasis of B16F10-GFP melanoma were subjected to bacterial treatment. As expected, a dramatic increase in the density of F4/80^+^KCs along with a reduction in GFP^+^ tumor areas were observed in the livers of *E*. *coli–*sg*Mafb/Maf*–treated mice as compared with *E*. *coli–*vector–treated control mice (Figure 5, A–C). Most of these KCs were also positive for TIM4, a discriminating marker for tissue-resident macrophages (Figure 5A), excluding the possibility that they were recently recruited monocyte-derived macrophages (37, 38). The expansion of F4/80^+^TIM4^+^ KCs was also validated by flow cytometry, showing an approximately 5-fold increase in these cells (Figure 5, D and E) and a striking upregulation of their Ki67 levels upon simultaneous editing of *Mafb/Maf* (Figure 5F). In contrast, editing *Mafb* or *Maf* alone was insufficient to provoke robust KC proliferation and expansion (Supplemental Figure 13, C–E). Notably, *E*. *coli–*sg*Mafb/Maf* injection did not amplify KCs in tumor-free naive mice (Supplemental Figure 13, F and G), indicating the involvement of tumor-derived factors in driving the proliferation of MafB/c-Maf–deficient KCs. The tumor microenvironment is known to be enriched with cytokines that promote macrophage infiltration and proliferation, including macrophage CSF (M-CSF), granulocyte macrophage CSF (GM-CSF), and IL-4 (39). We then measured the mRNA levels of these cytokines upon recombinant bacterial treatment. *Csf1*, but not *Csf2* or *Il4*, was highly expressed in tumors compared with tumor-distant liver tissues (Figure 5G), implying that enhanced local production of M-CSF may stimulate KC mobilization and proliferation. In support of this, we showed that proliferating KCs, as marked with TIM4^+^RFP^hi^ cells in *Ki67*-RFP reporter mice, closely abutted the edge of liver tumors early after bacterial therapy, but were barely seen in tumor-distant liver tissues (Figure 5H). Blockade of M-CSF signaling using anti-CSF1R antibodies severely diminished the number of proliferating KCs in tumor-adjacent areas, leading to impaired expansion of resident KCs upon *E*. *coli–*sg*Mafb/Maf* treatment (Figure 5, I and J). These data thus indicate that KC proliferation primarily occurred in peritumoral regions in response to local production of M-CSF.

**Figure 6:**
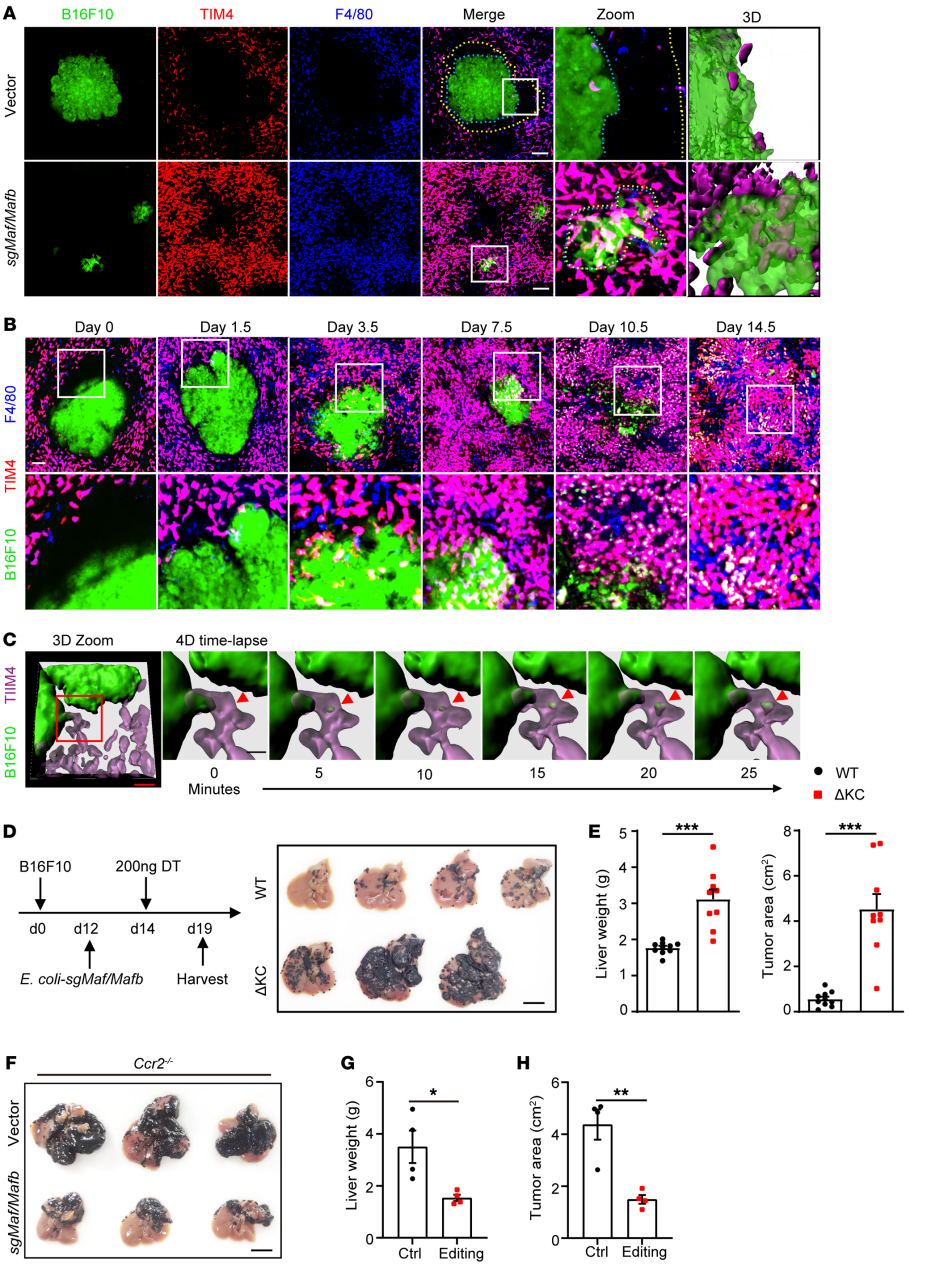
KC-dependent elimination of liver metastasis during BIL-CRISPR–mediated bacterial therapy. (**A**) Mice with established B16F10 liver metastasis were treated with *E*. *coli*–vector or *E*. *coli*–*sgMafb/Maf* i.v. Representative intravital liver images at 7 days after bacterial treatment are shown. Scale bars: 100 μm. Original magnification, zoomed images: × 3.8. Peritumoral KC dark zones are show between yellow and blue dashed lines. (**B**) Intravital liver images of tumor-KC interfaces at various time points after bacterial treatment. Scale bars: 100 μm. Original magnification, zoomed images: × 2.9. (**C**) Representative time-lapse 3D intravital liver images showing KCs nibbling an interacting cancer cell after bacterial therapy. Scale bars: 40 μm (red); 15 μm (black). (**D**) WT or *Clec4f*-iDTR mice with established B16F10 liver tumors were treated with DT 2 days after ClearColi *sgMafb/Maf* treatment, and the livers were harvested 5 days later, as depicted. Scale bars: 1 cm. (**E**) Liver weights and tumor area on the surface of livers were measured in **D**. *n* = 9–10 mice per group pooled from 3 independent experiments. (**F**) *Ccr2^–/–^* mice with established B16F10 liver tumors were injected with ClearColi-vector or ClearColi-*sgMafb/Maf* at day 12 and harvested at day 19. (**G** and **H**) Liver weights and the tumor area on the surface of livers from **F** were measured. *n* = 4 mice from 1 of 2 independent experiments. Data are represented as mean ± SEM. *P < 0.05; **P < 0.01; ***P < 0.001, unpaired Student’s *t* test.

In addition to KC expansion, bacterial therapy also led to the disappearance of the peritumoral KC dark zone in tumor-bearing livers (Figure 6A). 3D reconstruction revealed that GFP^+^ metastatic tumors were immersed in KC-rich areas, allowing easy access of KCs to cancer cells (Figure 6A and Supplemental Figure 14). We imaged the KC-tumor interface at various time points after *E*. *coli–*sg*MafB/c-Maf* injection, and F4/80^+^TIM4^+^ tissue-resident KCs were found to significantly accumulate at the tumor border over time (Figure 6B). Some of these cells penetrated the tumor core and ingested GFP particles a few days after bacterial therapy, suggesting active uptake of cancer cells (Figure 6B). In fact, time-lapse intravital imaging showed that tumor-infiltrating KCs can extend cell protrusions to intimately interact with the tumor and grab a piece of cell fragment from the contacting cancer cell (Figure 6C and Supplemental Video 4). This nibbling behavior seemed to be predominant in KC-mediated elimination of cancer cells during therapy, as we did not observe any KCs that engulfed a whole tumor cell at all time points examined. As a result, macrometastases were gradually disrupted and digested, with no substantial GFP^+^ tumors remaining by day 10.5 after therapy. At this time, KCs abutting the tumor foci had almost entirely ingested GFP^+^ contents from cancer cells (Figure 6B). Selective depletion of KCs after *E*. *coli–*sg*Mafb/Maf* injection completely abolished the bacteria-mediated antitumor effect (Figure 6, D and E), highlighting the importance of resident KCs in eliminating metastatic cancers. In contrast, *Ccr2^–/–^* tumor-bearing mice remained sensitive to *E*. *coli–*sg*MafB/c-Maf* treatment, indicating that monocytes and their macrophage derivatives were dispensable for this profound antitumor effect (Figure 6, F–H). Taken together, these findings suggested that BIL-CRISPR–mediated in situ editing of *Mafb*/*Maf* induced massive KC proliferation, intratumoral infiltration, nibbling of cancer cells, and dismantling of large tumors, collectively contributing to rapid tumor regression.

### Bacterial therapy skewed the polarization of KCs toward proinflammatory and induced robust T cell immunity.

**Figure 7:**
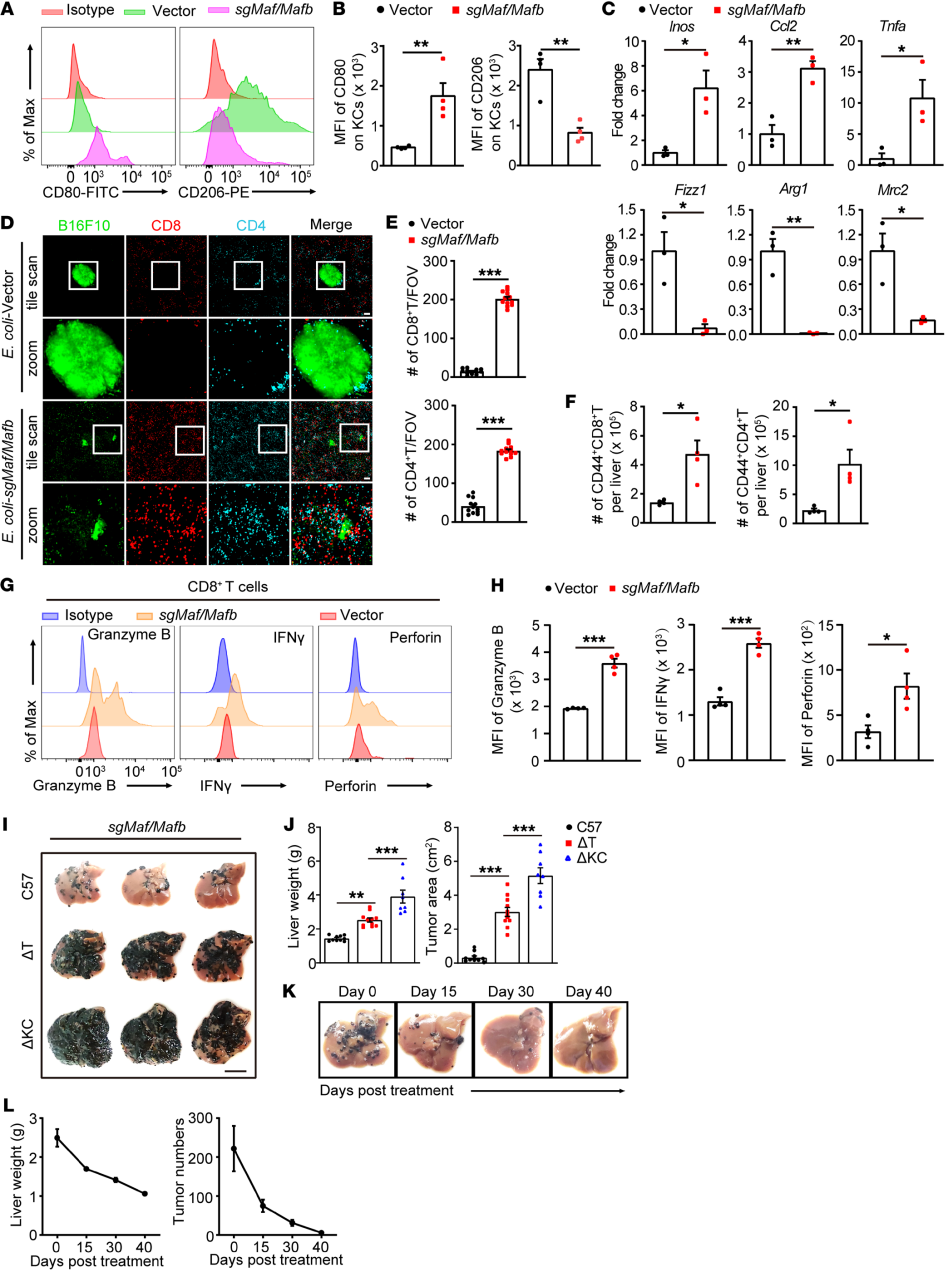
Reshaping the tumor microenvironment by BIL-CRISPR–mediated bacterial therapy. (**A**) Mice with established B16F10 liver metastasis were treated with *E*. *coli*–vector or *E*. *coli*–*sgMafb/Maf* for 7 days. Representative flow cytometric plots of CD80 and CD206 expression on KCs are shown. KCs were pregated as CD45^+^Ly6G CD11b^lo^F4/80^hi^TIM4^+^ cells. (**B**) MFI of CD80 and CD206 on KCs was quantified. *n* = 4 mice. (**C**) Normalized mRNA expression of *Inos*, *Ccl2*, *Tnfα*, *Fizz1*, *Arg1*, and *Mrc2* in KCs sorted from bacteria-treated tumor-bearing mice. *n* = 3 mice. (**D**) Representative liver images showing tumor infiltration of T cells 7 days after bacterial treatment. Scale bars: 100 μm. Original magnification, zoomed images: × 3.0. (**E**) Number of CD4^+^ and CD8^+^ T cells per FOV in **D**; a total of 12 FOVs from 3 mice per group were analyzed. (**F**) Number of hepatic CD44^+^CD4^+^ and CD44^+^CD8^+^ T cells measured by flow cytometry. *n* = 4 mice. (**G**) Representative histogram and (**H**) MFI of granzyme B, IFN-γ, or perforin expression in hepatic CD8^+^ T cells from *E*. *coli*–vector or *E*. *coli*–*sgMafb/Maf* treated mice. *n* = 4 mice. (**I**) Sex- and age-matched WT, *CD4*-iDTR, or *Clec4f*-iDTR mice with established B16F10 liver tumors were treated with DT at day 2 after ClearColi-*sgMafb/Maf* injection, and the livers were harvested at day 7. Scale bar: 1 cm. (**J**) Liver weights and tumor area in **I** were measured. *n* = 8–11 mice. (**K**) B16F10 tumor-bearing mice were treated with *E*. *coli–sgMafb/Maf*. Livers were harvested at the indicated time points after bacterial treatment, and representative liver pictures are shown. (**L**) Liver weights and number of hepatic tumor nodules were measured. *n* = 3–4 mice for each time point. Representative or pooled data from 2 independent experiments are shown. Data are represented as mean ± SEM. *P < 0.05; **P < 0.01; ***P < 0.001, Student’s *t* test (**B**, **C**, **E**, **F** and **H**); 1-way ANOVA with Tukey’s test in (**J**).

Both MafB and c-Maf were reported to promote antiinflammatory macrophage polarization (40, 41). We thus hypothesized that bacteria-mediated genetic inactivation of these transcription factors could skew the polarization states of KCs in liver metastasis. Indeed, whereas KCs in control tumor-bearing mice exhibited a typical CD80^lo^CD206^hi^ alternative-activated macrophage phenotype, KCs in *E*. *coli–*sg*Mafb/Maf*-treated mice were CD80^hi^CD206^lo^, representing proinflammatory macrophages (Figure 7, A and B). In line with this, *Mafb*/*Maf* editing induced a strong regulation of M1-associated genes (*Inos*, *Ccl2*, and *Tnfa*) and concomitant downregulation of M2-associated genes (*Fizz1*, *Arg1*, and *Mrc2*) in KCs of tumor-bearing mice (Figure 7C). Consequently, the T cell–unfavorable tumor microenvironment in liver metastasis (4) was reshaped, as evidenced by abundant intratumoral infiltration of CD4^+^ and CD8^+^ T cells in *E*. *coli–*sg*Mafb/Maf*–treated mice compared with *E*. *coli–*vector–treated mice (Figure 7, D and E, and Supplemental Figure 15, A and B). Meanwhile, the majority of these T cells showed an activated CD44^hi^ phenotype (Figure 7F), with significantly enhanced production of effector molecules, such as IFN-γ, granzyme B, and perforin in CD8^+^ T cells (Figure 7, G and H). To assess the contribution of T cells to tumor regression, we used *Cd4*-iDTR mice to enable inducible ablation of T cells during bacterial treatment (Supplemental Figure 15, C–F). T cell depletion severely diminished *E*. *coli*–sg*Mafb/Maf*–mediated therapeutic effects against liver metastasis; however, the tumor burden in T cell–depleted mice remained significantly lower than that in KC-depleted mice (Figure 7, I and J). These data supported an indispensable role of T cells in tumor regression, but also suggested that KC-mediated tumoricidal activity was not solely dependent on T cells. We speculated that the induction of robust T cell responses could be beneficial for the long-term control of liver metastasis. In fact, no tumor relapse was observed in the liver by day 40 after bacterial therapy (Figure 7, K and L). Taken together, our bacteria-based immunotherapy reshaped the tumor immune microenvironment, leading to efficient and durable antitumor immunity against liver cancer.

### Disruption of MafB/c-Maf enhanced the antitumor activity of human macrophages upon M-CSF and bacterial treatment.

**Figure 8:**
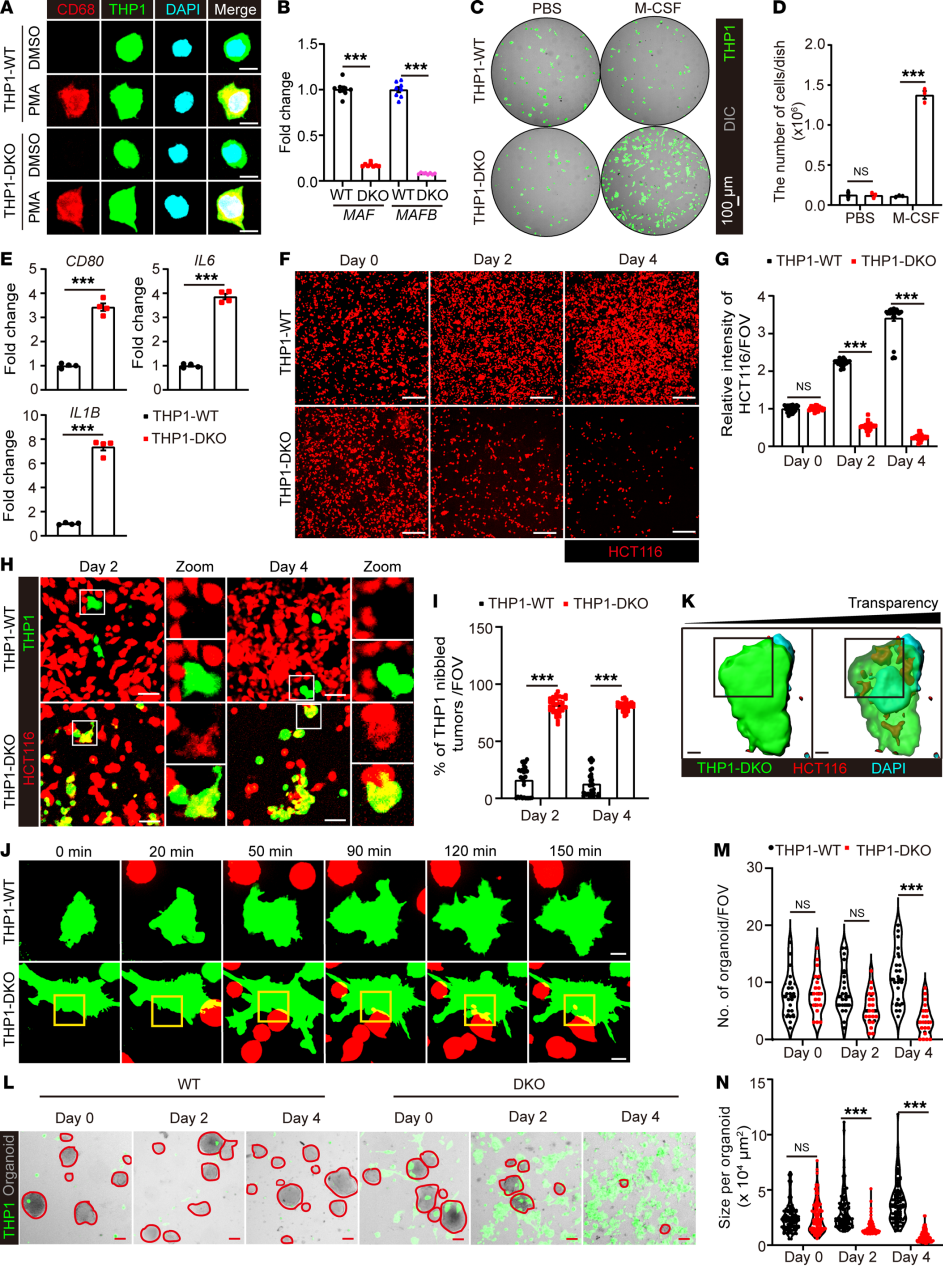
Enhanced antitumor activity of human macrophages by c-Maf/MafB inactivation during M-CSF and bacterial treatment. Validation of ZsGreen-expressing WT or DKO THP1-derived macrophages by immunofluorescent staining of (**A**) hCD68, and (**B**) qPCR detection of *MAF* and *MAFB* expression. *n* = 8 cell samples. Scale bars: 10 μm. (**C**) Representative images of THP1-WT or DKO macrophages cultured in the absence or presence of human M-CSF for 3 days. (**D**) Quantification of cell numbers in **C**. *n* = 4 cell samples. (**E**) THP1 macrophages were treated with ClearColi for 8 hours and washed away. Expression of typical human M1 macrophage–associated genes (*CD80*, *IL6*, *IL1B*) was detected at 48 hours. *n* = 4 cell samples. (**F**) Representative images of HCT-116-tdTomato cells at days 0, 2, and 4 after coculture with hM-CSF and ClearColi primed THP1-WT or DKO macrophages. Scale bars: 200 μm. (**G**) HCT-116 cell density per FOV measured by tdTomato fluorescence intensity in **F**. *n* = 30 FOVs. (**H**) Representative images of macrophage and HCT116 tumor cell coculture at the indicated time points. Scale bars: 50 μm. Original magnification, zoomed images: × 2.5. (**I**) Percentages of macrophages with tumor fragments inside the cells. *n* = 30 FOVs. (**J**) Live-cell imaging and (**K**) 3D reconstitution of macrophage-tumor interactions during coculture. Scale bars: 10 μm. (**L**) Representative images of macrophages and patient-derived CRC organoid coculture at indicated time points. Scale bars: 100 μm. (**M**) Number and (**N**) size of organoids in **L** were measured. *n* = 20 FOVs. Representative or pooled data from 2 independent experiments are shown. Data are represented as mean ± SEM. **P < 0.01; ***P < 0.001, 2-way ANOVA with Tukey’s test (**B**, **D**, **G**, **I**, **M** and **N**); Student’s *t* test in (**E**).

We next tested to determine whether disruption of MafB/c-Maf expression could enhance the antitumor activity of human macrophages in vitro to provide support for the translational potential of our approach. Because of the notorious difficulty of genetic manipulation of multiple genes in primary human macrophages, we developed a ZsGreen-expressing, Mafb and c-Maf double-deficient (DKO) THP-1 cell line and differentiated these cells into human macrophages as an alternative (Figure 8, A and B). In line with our in vivo findings, DKO macrophages propagated robustly in the presence of human M-CSF (hM-CSF) and polarized toward a proinflammatory phenotype after phagocytosing ClearColi (Figure 8, C–E, and Supplemental Figure 16, A–E). We then examined the tumoricidal capacity of these DKO macrophages against HCT116 colon cancer cells, which represent the most common type of cancer that undergoes liver metastasis. Interestingly, the number of tdTomato-expressing HCT116 tumor cells declined drastically after coculture with ClearColi-primed DKO but not WT macrophages in the presence of hM-CSF (Figure 8, F and G), coinciding with a profound amplification of DKO macrophages and significant accumulation of tumor cell corpses inside these cells (Figure 8, H and I, and Supplemental Figure 16F). We then performed live-cell imaging to track the dynamic interactions between macrophages and tumor cells. While whole-cell engulfment was rarely observed, DKO macrophages were frequently found to grab small cell fragments from the body of intimately interacting tumor cells (Figure 8, J and K, and Supplemental Video 5), resembling the tumor-nibbling behavior of *MafB/Maf*-edited KCs in vivo. To further validate the antitumor function of DKO macrophages against human cancers, we generated patient-derived CRC organoids and monitored their growth during coculture with macrophages. Consistently, both the number and size of tumor organoids were significantly reduced in the presence of ClearColi plus hM-CSF–primed DKO macrophages (Figure 8, L–N). 3D reconstruction analysis also revealed that DKO but not WT macrophages can penetrate into and internalize bites from interacting tumor organoids (Supplemental Figure 16, G and H). These data collectively implied that bacteria-mediated genetic inactivation of Mafb and c-Maf in KCs may hold therapeutic promise against human cancers.

## Discussion

As the most abundant immune cells in the liver, KCs, when dysfunctional, contribute to the immunopathogenesis of various types of liver diseases. Modulating KC activity represents an attractive therapeutic approach against liver pathologies. Here, we report a simple and economical strategy for genetically modifying and functionally reprogramming KCs in vivo. This was achieved by utilizing attenuated bacteria as KC-targeting plasmid DNA delivery vehicles. Circulating bacteria were rapidly captured by KCs and were subsequently enclosed in phagosomes (42). Plasmid transfer from these bacteria into the cytosol and nuclei of KCs was required for the successful expression of exogenous DNA. Unlike intracellular bacteria, such as *Listeria monocytogenes*, which can escape phagosomes by secreting pore-forming toxins (43), the *E*. *coli* we used has no such immune-evasion strategy. Given that efficient gene delivery only occurred when KCs caught a large number of *E*. *coli*, one could speculate that the phagolysosome compartment of KCs and its bactericidal ability were saturated under this condition, resulting in the escape of *E*. *coli* from intracellular vacuoles into the cytosol, where the bacteria were lysed and plasmids were released. In support of this hypothesis, a substantial proportion of bacteria remained alive inside KCs even at day 3 after high-dose *E*. *coli* infection, and complete eradication of these bacteria took more than 7 days, a time period that was longer than what we expected based on our experience with low-dose *E*. *coli* infections. This delayed bacterial clearance may reflect inefficient bacterial killing in the phagolysosome of KCs. Moreover, *E*. *coli* expressing listeriolysin O (LLO), a pore-forming toxin that facilitates bacterial escape from phagolysosomes (44), did not further increase the efficacy of plasmid DNA delivery to KCs (our unpublished data). Therefore, it was very likely that bacterial lysis in the cytosol rather than bacterial escape from vacuoles was a bottleneck for efficient gene delivery in KCs. In this regard, engineering *E*. *coli* to express suicide gene elements that induce the self-lysis of bacteria inside KCs may further improve the gene-editing efficacy and safety of our current method (45). This would be important for future applications because some genes (e.g., *Timd4*) seemed to be more resistant to BIL-CRISPR–mediated gene deletion than others (e.g., *Crig*, *Maf*, etc.). Increasing the intranuclear concentration of plasmid DNA in KCs, i.e., increasing the availability of sgRNAs and Cas nucleases, may help in deleting these resistance genes.

KCs are probably the macrophage population with the highest phagocytic capacity in the body. Specific depletion of KCs using *Clec4f*-iDTR mice before or after the onset of liver metastasis suggested that KC dysfunction was a key determinant of metastatic liver cancer progression. One of the underlying mechanisms we reported here could be the inaccessibility of KCs to cancer cells at the late stage of liver metastasis. Why and how the peritumoral KC “dark zone” was formed remain to be investigated, but its appearance was associated with an overall reduction in KC cell number in tumor-bearing mice, suggesting preferential KC loss in peritumoral areas. Recent studies have raised the possibility that KC loss is a general phenomenon that occurs in various liver injuries (46), which may also apply to liver metastasis because metastatic tumor growth inevitably inflicts host tissue damage, particularly in tumor-adjacent areas. KCs residing in those areas may sense tumor-induced collateral tissue damage and undergo cell death. Depletion of tissue-resident KCs resulted in an open macrophage niche in the liver, which provoked hepatic infiltration and differentiation of monocyte-derived macrophages (31, 47, 48). Whether the loss of peritumoral KCs represents an immune evasion strategy that facilitates the recruitment and development of bone marrow–derived macrophages to exert tissue remodeling and other protumoral functions merits further investigation.

We showed that liver metastasis-induced KC loss and dysfunction can be overcome by disruption of MafB/c-Maf expression in KCs, leading to massive proliferation and intratumoral infiltration of TIM4^+^ tissue-resident KCs. Of note, c-Maf and MafB were not uniformly expressed in KCs, and a proportion of KCs with self-renewing capacity had much lower levels of c-Maf or MafB expression than others under steady state (36). Why did these cells not proliferate during liver metastasis to maintain the KC pool in response to tumor-derived M-CSF? A possible reason was that these self-renewing KCs were more prone to tissue injury–induced cell loss and were depleted in tumor-adjacent areas. The mechanisms underlying KC mobilization have not been elucidated, but tumor-derived macrophage chemoattractants, such as M-CSF and CCL2, could be a clue. Depletion of MafB/c-Maf could alter the molecular pattern that regulates KC retention and migration, leading to their intratumoral infiltration under the guidance of tumor-enriched chemoattractants. In fact, KCs exhibited synchronized repolarization from M2 macrophages toward M1 macrophages during our bacterial therapy. These 2 different types of macrophages are known to have distinct migration abilities, which act by expressing different adhesion molecules (49, 50). In addition to MafB/c-Maf inactivation, bacterial stimulation may also be a determinant for the M2-to-M1 transition of KCs. Although the *E*. *coli* we used were disabled in triggering LPS-related inflammatory responses, bacteria-derived DNA or RNA molecules can act as pattern recognition molecular patterns (PAMPs) to stimulate proinflammatory signaling cascades in KCs. For instance, activation of the cGAS/STING pathway by cytosolic DNA is known to direct M1 macrophage polarization and facilitate antitumor immunity (51). In this regard, the combination of MafB/c-Maf inactivation and bacterial stimulation jointly reprogrammed KC function in liver metastasis. Further studies using mice deficient in STING or other cytosolic DNA/RNA sensors/signaling adaptors could aid in addressing the molecular mechanisms underlying KC repolarization.

Once entering the tumor, Mafb/cMaf-inactivated KCs unleashed an incredible tumoricidal response contributed by both KC- and T cell–mediated tumor killing. Rather than engulfing a complete cancer cell, KCs were found to intimately adhere to the metastatic tumor using their cell protrusion and seize a small piece from the interacting cancer cell. This nibbling behavior was strongly reminiscent of trogocytosis, which was preferentially used by macrophages to destroy objects that were too large to be phagocytosed, such as presynaptic structures (52). This could also hold true for macrophages to eliminate cancer cells, which are usually large in size. In fact, we found that, although KCs were able to arrest circulating tumor cells, they did not completely engulf these cells. Instead, the arrested tumor cells seemed to be ripped off by KCs, and their cell fragments were subsequently internalized. This was consistent with a previous study showing that KCs were unable to phagocytose a complete cancer cell unless in the presence of antibody opsonization (53). These data therefore pointed out the potent tumor-killing ability of KCs, which was very likely mediated by trogocytosis-induced cell death (54, 55). Given that T cell depletion also severely dampened the therapeutic effects of recombinant bacterial therapy, the extent to which KC-mediated direct killing of cancer cells contributed to tumor regression in our model was unclear. We inferred that the persistent nibbling/trogocytosis of tumors by KCs can cumulatively dismantle the T cell–exclusionary macrometastases into micrometastases that are permissive for T cell infiltration and killing. Therefore, enhancing macrophage trogocytosis may represent an efficient strategy for improving the efficacy of current immunotherapy to combat “cold” tumors. However, due to the limited resolution of in vivo imaging, we cannot discriminate at this stage between the behaviors of trogocytosis and efferocytosis of apoptotic bodies that were released from dying cancer cells, and further studies validating the role of trogocytosis in KC-mediated antitumor function are warranted.

In addition to mouse studies, we have set up an in vitro macrophage-tumor coculture system to demonstrate the increased tumoricidal activity of THP1-derived DKO macrophages against a human colon cancer line and patient-derived CRC organoids, confirming the superior capacity of Mafb/cMaf-inactivated human macrophages in nibbling and killing tumors upon M-CSF and ClearColi stimulation. Nevertheless, several limitations remain regarding the clinical relevance of this study. First, the THP1-derived human macrophages do not fully recapitulate the immune characteristics of human primary KCs. Second, this coculture system does not reflect the function of DKO macrophages in reshaping the tumor microenvironment and thus may underestimate their capacity to eliminate liver metastasis in vivo. Finally, the composition of liver myeloid cells in humans is different from that in mice. Some types of phagocytes, such as monocyte-derived macrophages, can even outnumber resident KCs in some individuals (33, 56). The specificity and efficiency of recombinant bacteria in targeting and editing resident human KCs in vivo remain to be determined. Even so, these in vitro studies provide evidence to support the notion that genetically modified human macrophages with MafB/cMaf deficiency can possess potent antitumor function, which will have great implications for designing macrophage-based adoptive cell therapy, such as chimeric antigen receptor (CAR) macrophages (57, 58).

Overall, we have developed an engineered bacteria-based approach to genetically modifying and functionally reprogramming KCs in situ. Targeted disruption of MafB and c-Maf expression using this approach could overcome tumor-induced KC loss and dysfunction, eliciting unprecedented therapeutic effects against various types of metastatic liver cancer in mice. Our study thus sheds light on the application of bacterial tumor therapy and could have translational potential for treating end-stage liver metastasis.

## Methods

### Mice.

C57BL/6 mice were purchased from Shanghai SLAC Laboratory Animal Co. *Clec4f*-Cre-tdTomato, iDTR, *Ki67*-RFP, Ai14, *Cd4*-Cre, *Ccr2*-RFP KI/KO, and *Ccr2*-CreERT2 (59) mice were originally from the Jackson Laboratory. *Clec4f*-CreERT2 mice were generated by Shanghai Model Organisms Center Inc. using CRISPR/Cas9-mediated homologous recombination. *Crig^–/–^* mice were generated by CRISPR/Cas9-mediated deletion of exons 1 and 2 of the *Crig* gene using 2 sgRNAs. All mice were maintained in a specific pathogen–free facility at USTC.

### Animal models.

A mouse model of liver metastasis was established by intrasplenic injection of tumor cells. Briefly, 6- to 8-week-old male mice were anesthetized by isoflurane, the spleen was exposed by a small incision in the left flank, and tumor cells were injected intrasplenically using a 30-gauge needle, followed by splenectomy 5 minutes after injection. Unless otherwise stated, a total of 3 × 10^5^ B16F10 cells, 3 × 10^5^ B16F10-ZsGreen cells, 1 × 10^6^ MC38 cells, or 2 × 10^6^ LLC cells were injected in 50 μL PBS, and tumor-bearing mice were sacrificed at day 15 after tumor injection. To study the therapeutic effects against late-stage liver metastasis, mice were treated with bacteria at day 12 after tumor injection and were sacrificed at day 19. Mice were monitored daily during this period, and an increase in body weight over 20% was considered as reaching the end point of the experiment. To establish ConA-induced inflammatory hepatitis, mice were injected i.v. with 15 mg/kg ConA (MilliporeSigma), and liver and serum samples were harvested at 24 hours after injection. To deplete KCs, *Clec4f*-iDTR mice were i.p. injected with 200 ng DT (MilliporeSigma, D0564), as illustrated in Figure 3 and Figure 6. To deplete T cells, *Cd4*-iDTR mice were i.p. administrated with 100 ng DT daily for 3 consecutive days. To block M-CSF signaling, mice were i.p. injected with 500 μg of control IgG or anti-CSF1R (ASF98, Bio X Cell) antibody at days 6, 8, and 10 after tumor inoculation. In fate-mapping experiments, a single dose or 3 consecutive doses of tamoxifen (1 mg, MilliporeSigma) were administered into *Clec4f*-CreERT2:Ai14 mice or *Ccr2*-CreERT2:Ai14 mice, respectively, by oral gavage before bacterial treatment.

### Bacteria and infection.

To generate fluorescence-tagged *E*. *coli*, pCM29-sfGFP (42) or pUC18T-mCherry (60) plasmids were transformed into TOP10 (TransGen Biotech) or ClearColi (Lucigen), respectively. GFP-tagged *S*. *aureus* was generated by electroporating pAT18-cGFP plasmids (61) into USA400 MW2 using RN4220 as a shuttle strain. For *E*. *coli* infection, TOP10 or ClearColi was grown in lysogeny broth (LB) medium for 16 to 18 hours at 37°C with shaking in the presence of appropriate antibiotics. Bacteria were then harvested, washed, and injected i.v. into mice at the indicated doses. For *S*. *aureus* infection, GFP-tagged MW2 cells were cultured overnight in brain heart infusion (BHI) medium with 5 μg/mL erythromycin, followed by subculture until reaching logarithmic phase (OD_660_ nm = 1.0). A total of 5 × 10^7^ CFU bacteria were injected i.v. into mice. Bacteria-infected mice were monitored for symptoms of sepsis as previously described (24). To evaluate the bacterial burden in organs, tissue homogenates were serially diluted in PBS and plated onto agar medium. The CFUs were enumerated 16 to 24 hours after incubation at 37°C.

### Plasmids and in vivo KC editing.

To construct a dual sgRNA expressing the CRISPR/Cas9 vector, the pX459 vector (Addgene, 62988) was first modified by replacing its puromycin fragment with a Cre-encoding sequence. A multiple cloning site containing HindIII/XhoI/BamHI/SalI was ligated between the original XbaI and KpnI restriction sites. The DNA fragment encoding the U6-BsaI-gRNA scaffold was cloned and then inserted into the vector backbone using XhoI- and KpnI-restricted enzymes, followed by introducing a second sgRNA expression cassette (U6-SapI-gRNA scaffold) via seamless DNA cloning. A similar strategy was used to construct a dual sgRNA expressing the CRISPR/CasΦ vector on the basis of the pPP441 (Addgene, 158801) vector. The resultant pX459-2U6-BsaI-SapI and pPP441-2U6-BasI-SapI vectors were used for in vivo genome editing of KCs. Briefly, 2 pairs of sgRNA oligos targeting different sites of a given gene were designed, synthesized, annealed, and ligated sequentially into dual sgRNA vectors using BsaI- and SapI-restricted enzymes. Sequence-verified plasmids were then electroporated into *E*. *coli* TOP10 or ClearColi, followed by plating of these transformed *E*. *coli* onto LB agar medium in 15 cm petri dishes with 100 μg/mL ampicillin (MilliporeSigma). After overnight culture, the *E*. *coli* bacteria were collected with cell scrapers, weighed, and washed. We quantified *E*. *coli* based on the cell weight as follows: total CFU = 30 × bacterial weight (g) ×10^10^. This calculation was verified many times in the lab and was more reliable than the OD measurement in our laboratory. For KC editing, a total of 10^9^ CFU *E*. *coli*–containing gene-targeting plasmids or backbone vectors were injected i.v. into mice via the tail vein in 200 μL saline. The gene-editing efficiency was detected 7 days later via intravital imaging of mouse liver or via RT-PCR detection of sorted KCs. The sequences of sgRNAs used in this study were as follows: *Crig*-exon1 (Cas9): TGAGCACTATTAGGTGGCCC; *Crig*-exon2 (Cas9): GGTCTCCAGTGGAGTCACGT; *Crig*-exon1 (CasΦ): CTGGGCCACCTAATAGTG; *Crig*-exon2 (CasΦ): CTACGTGACTCCACTGGA; *Timd4*-exon1 (Cas9): GCTCCGTCACCAGCCAGAGG; *Timd4*-exon2 (Cas9): GGTGTACTGCTGCCGTATAG; *Adgre1*-exon3 (Cas9): TATTACTGCACCTGTAAACG; *Adgre1*-exon4 (Cas9): GCCAAGTGCAGCTGTCTTAG; *Mafb*-exon1-1 (CasΦ): TCAAGTTCGACGTGAAGA; *Mafb-*exon1-2( CasΦ): GCGAGTTTCTCGCACTTG; *Maf*-exon1-1 (CasΦ): CGACCTGCCCACCAGTCC; *Maf*-exon1-2 (CasΦ): TCTCGGAAGCCGTTGCTC; *Rosa26*-exon1 (CasΦ): GGGGCTCCGGCTCCTCAG; *Rosa26*-exon2 (CasΦ): CTGCTGTCTGAGCAGCAA; *Trem1*-exon2-1 (Cas9): AGCACAACAGGGTCATTCGG; and *Trem1*-exon2-2 (Cas9): GCAGAGACTACCAGACGGGA.

### IVM.

Spinning disk confocal intravital imaging of mouse liver was performed as previously reported (23, 24). Briefly, mice were anesthetized by 2.5% avertin (300 mg/kg/mouse, containing 0.25 g 2,2,2-tribromethano [MilliporeSigma] and 0.25 mL 2-methyl-2-butanol [MilliporeSigma]). Tail-vein cannulation was conducted to permit the delivery of fluorescently labeling reagents and additional anesthetics. The left lateral lobe of the liver was externalized onto a glass coverslip embedded in a customized sample holder of the microscope and covered with strips of saline-moistened Kimwipes (Fisher Scientific) to restrict movement. The stage was heated to maintain a body temperature of 37°C in mice. Images were then acquired using an inverted microscope (Nikon Ti2-E) equipped with a Yogokawa CSU-W1 Spinning Disk Confocal Scanner Unit. Fluorescence was visualized with the iChrome MLE compact 4-color laser engine (405 nm, 488 nm, 561 nm, and 640 nm; Toptica) coupled with 4 emission filters (B447/60, B525/50, B617/73, and B685/40) and was recorded with an sCMOS camera (Prime95B, Photometrics) offering a large field of view (a sensor diagonal of 18.7 mm) and a high resolution (11 μm pixel size). Imaging data were analyzed using NIS-Elements AR software (version 5.20.00) or ImageJ (Fuji). 3D reconstruction was conducted using Imaris (version 7.0, Bitplane).

### Flow cytometry.

For isolation of liver nonparenchymal cells (LNPCs), mouse livers were harvested, minced into small pieces, and then dissociated by GentleMACs. The liver homogenates were digested in prewarmed DMEM containing 0.5 mg/mL collagenase I (MilliporeSigma) and 5 U/mL DNase I (MilliporeSigma) at 37°C for 20 minutes under shaking, followed by filtering through a 200-gauge mesh. After a short centrifugation to remove hepatocytes and tissue debris, cells were pelleted and washed by centrifugation at 400*g* for 5 minutes at 4°C. LNPCs were then obtained after lysis of red blood cells in ACK buffer (BioLegend) and resuspended in ice-cold 1× PBS. For flow cytometry, a total of 1 × 10^6^ cells were incubated with Fc blocker (2.4G2, Bio X Cell) for 20 minutes and then stained with a mixture of fluorophore-conjugated antibodies against the surface markers for 30 minutes at 4°C in the dark. For intracellular staining, surface marker–stained cells were fixed and permeabilized using a Foxp3 staining buffer set (eBioscience), followed by staining with Ki67 or other intracellular molecules. For cytokine detection, cells were pretreated with BFA (5 μg/ml) for 4 hours before intracellular staining. DAPI (Biosharp) or Zombie Violet dyes (BioLegend) were used to distinguish live/dead cells. Cell-counting beads (BioLegend) were added to determine the absolute cell numbers of the samples. Flow cytometry was conducted using either BD LSRFortessa or Beckman CytoFLEX. For sorting of tissue-resident KCs, LNPCs were isolated, treated with Fc blocker, and stained with fluorescent-conjugated antibodies. DAPI^–^CD45^+^TIM4^+^ cells were sorted using BD FACSAria III with a purity higher than 95%.

### Statistics.

All experiments were repeated independently at least twice with 3 to 5 biological samples per group. Statistical analysis was carried out using GraphPad Prism, version 9.0. Data were expressed as the mean ± SEM. Unpaired Student’s *t* test was used for comparisons between 2 individual groups. One-way or 2-way ANOVA with Tukey’s test was used for multiple group comparisons. Mouse survival was analyzed using a 2-sided log-rank test. A *P* value of less than 0.05 was considered significant.

### Study approval.

Animal experiments were performed under the guidelines established by the animal care committee of USTC with the approval number USTCACUC192401034. Clinical samples were collected with written, informed consent obtained from patients. All related studies were approved by the Institutional Review Board of USTC.

A more detailed description of the materials and methods is provided in the Supplemental Methods.

## Author contributions

WL and XZ designed and conducted the experiments and analyzed the data. QY, CC, QZ, KD, and LL performed some experiments. ZZ designed the research, supervised the study, and wrote the manuscript. The order of co–first authors was determined based on their time spent on this project.

## Supplementary Material

Supplemental data

Supplemental video 1

Supplemental video 2

Supplemental video 3

Supplemental video 4

Supplemental video 5

## Version 1 02/23/2023

In-Press Preview

## Version 2 04/17/2023

Electronic publication
