# Supplementary material for: In situ expansion and reprogramming of Kupffer cells elicit potent tumoricidal immunity against liver metastasis
Source: J Clin Invest. 2023 Apr 17;133(8):e157937. doi: 10.1172/JCI157937 (PMC10104906; doi:10.1172/JCI157937)
Supplement: Supplemental data [file jci-133-157937-s154.pdf]

Supplementary materials for

In situ expansion and reprogramming of Kupffer cells elicits potent tumoricidal immunity against liver metastasis

Wei Liu[1*], Xia Zhou[1*], Qi Yao[1,2], Chen Chen[1,2], Qing Zhang[1,2], Keshuo Ding[3], Lu Li[1], Zhutian Zeng[1,2, #]

1. Department of Oncology, The First Affiliated Hospital of USTC, Division of Molecular Medicine, University of Science and Technology of China, Hefei, 230001, China,

2. The CAS Key Laboratory of Innate Immunity and Chronic Disease, School of Basic Medical Sciences, University of Science and Technology of China, Hefei, China

3. Department of Pathology, School of Basic Medicine, Anhui Medical University, Hefei, Anhui, 230032, China; Department of Pathology, the First Affiliated Hospital of Anhui Medical University, Hefei, Anhui, 230022, China

* Co-first authors

# Corresponding should be addressed to zengzt@ustc.edu.cn

This file includes

Supplemental Methods

Captions for Movies 1 to 5

Supplementary Figures and Figure legends 1 to 16

Supplemental Methods

Cell lines. B16F10, LLC, 293T, HCT116 and THP1 cells were purchased from the China Center of Type Culture Collection (CCTCC). The MC38 cell line was obtained from Dr. Zhigang Tian (USTC, Hefei). The B16F10-ZsGreen, LLC-ZsGreen, THP1-ZsGreen and HCT116-tdTomato cell line were generated by lentivirus transfection, and stably transfected cells were selected. DKO THP1 cells were generated by sequentially infecting THP1-ZsGreen cells with lentivirus-containing *MAFB* and *MAF* specific shRNAs followed by selection of stably-transfected cells. The target sequence for *MAF* was 5'-TGG AAG ACT ACT ACT GGA TGA-3', and that for *MAFB* was 5'-GCC CAG TCT TGC AGG TAT AA A-3'. All cell lines were cultured in RPMI1640 or Dulbecco's modified Eagle medium (Gibco) supplemented with 10% fetal calf serum (Biological Industries) and 1% penicillin/streptomycin.

Antibodies and reagents. Fluorophore-conjugated antibodies or reagents used for flow cytometry and intravital imaging were listed as following: AF700 anti-mouse CD45 (30-F11); APC/Cy7-conjugated anti-mouse CD3 (145-2C11); FITC anti-mouse CD8β (YTS156.7.7); PerCP/Cy5.5 anti-mouse CD8α (53-6.7); PE- anti-mouse CD4 (RM4-5); BV421 anti-mouse CD4 (GK1.5); PE/Cy7 anti-mouse NK1.1 (PK136); APC anti-mouse CD44, (IM7); APC/Cy7 anti-mouse CD45 (30-F11); PE/Cy7 anti-mouse CD11b (M1/70); PerCP/Cy5.5 anti-mouse Ly6C (HK1.4); AF700 anti-mouse LY6G (1A8); FITC anti-mouse CD80 (16-10A1); PE anti-mouse TIM4 (RMT4-54); AF647 anti-mouse TIM4 (RMT4-54); PE anti-mouse F4/80 (BM8); AF647 anti-mouse F4/80 (BM8); BV421 anti-

mouse F4/80 (BM8); FITC Armenian Hamster IgG Isotype Ctrl (HTK888); FITC anti-mouse KI67 (16A8); PE- anti-mouse CD206 (C068C2); FITC Annexin-V (640906); PerCP/Cy5.5 7-AAD (420403); Propidium Iodide solution (421301); PE Rat IgG2a, κ Isotype Ctrl (RTK2758); FITC Rat IgG2a, κ Isotype Ctrl (RTK2758), all these abovementioned antibodies or reagents were from Biolegend. AF488-, PE- or APC-conjugated anti-mouse CRIg (NLA14) was purchased from eBioscience. pHrodo™ Red (P36600) and anti-human MARCO (PA5-98947) were purchased from Invitrogen. PE anti-mouse secondary antibody (sc-3738) and FITC anti-rabbit secondary antibody (sc-2359) were purchased from Santa Cruz. PMA (P1585) was from Sigma. BFA (420601) was purchased from Biolegend. Human M-CSF (300-25) was purchased from Peprotech. For in vivo imaging of KCs, fluorophore-conjugated anti-F4/80 (1.8 μg), anti-TIM4 (2 μg) or anti-CRIg (2.5 μg) was injected into mice via tail vein cannulation immediately before surgical preparation for IVM of the liver. In some experiments, 50 mg/kg body weight FITC-dextran (MW150KDa, Sigma–Aldrich) or TRITC-dextran (MW155KDa, Sigma–Aldrich) was injected intravenously to visualize blood vessels.

Immunofluorescence. Paraffin-embedded liver sections from patients with colorectal liver metastasis were collected from the Department of Pathology, the First Affiliated Hospital of Anhui Medical University. These tissues were obtained from 6 patients who underwent surgical resection between 2019 and 2022. Sections were deparaffinized with xylene followed by graded ethanol treatment (100%, 95%, 90%, 80%, 70% and

50%) and rehydration. After antigen retrieval and blocking, sections were stained with anti-human-MARCO (1:100 dilution) overnight at 4 °C and followed by incubation with a mouse anti-rabbit secondary antibody (1: 300 dilution) at room temperature for 1 h. DAPI were used to label the nuclei. For immunofluorescence staining of in vitro cultured macrophages, THP1-differentiated-macrophages were fixed with PBS containing 0.2% Triton X-100 and 4% paraformaldehyde at room temperature for 10 mins. After blocking with 5% BSA for 30 mins, cells were stained with anti-human CD68 primary antibodies (Clone KP1, Santa Cruz, 1:100 dilution) overnight at 4 °C. Cells were then washed and incubated with goat anti-mouse secondary antibodies (1:500 dilution) at room temperature for 1 h, followed by DAPI counterstaining for 5-10 min.

Macrophage-Tumor coculture. THP1 cells were seeded on glass bottom dishes and differentiated into macrophages in the presence of 20 ng/ml PMA for 48 h. Differentiated THP1-WT or DKO macrophages (5×10^5 cells) were treated with 100 ng/ml human M-CSF for 3 days and stimulated with 5×10^7 CFU of Clearcoli during the last 8 h, followed by 3 steps of washing to remove free bacteria. A total of 5×10^5 HCT116 cells were then added to the macrophage culture and still images were taken at day 0, 2, and 4. Time-lapse imaging of macrophage-tumor coculture was acquired by an inverted microscope (Nikon Ti2-E) equipped with a Yogokawa CSU-W1 spinning disk confocal scanner and a live-cell microscope incubation system (Tokai Hit). Patient-derived tumor organoid lines were originally generated from treatment-free colorectal cancer patients and were kindly provided by Dr. Xiaoxing Li (Sun Yat-sen University

Cancer Center, Guangzhou). Organoids were seeded into Matrigel drops in 12-well plates (Corning) and cultured using advanced DMEM/F12 (Gibco) supplemented with 1% pen/strep, 10 mM HEPES (Gibco), 2 mM GlutaMAX (Gibco), 50% Wnt3a (RD), 20% R-spo1 (Peprotech), 1×B27 (Thermo), 1×N2 (Thermo), 10 mM nicotinamide (Sigma-Aldrich), 1.25 mM N-acetyl-l-cysteine (Sigma-Aldrich), 100 μg/ml primocin (Invivogen), 10 μM Y-27632 (Selleck chemicals), 100 ng/ml Noggin (Peprotech), 0.5 μM A83-01 (Tocris), 50 ng/ml EGF (Peprotech), 10 nM Gastrin (Genscript) and 3 μM SB202190 (Sigma-Aldrich). Intact organoids were released from Matrigel using TrypLE Express enzymes. Approximately 300 organoids were then added to the macrophage culture (primed with hMCSF and Clearcoli as depicted) containing 50% organoid culture medium, 50% RPMI 1640 (RPMI-GlutaMAX supplemented with 10% FBS and 1% pen/strep) and 5% Matrigel. Bright-field and fluorescent images were taken at the indicated time points.

Real-time RT PCR. RNA extraction and purification were carried out using either the RNeasy mini RNA isolation kit (Qiagen, Chatsworth, CA) or TRIzol reagent (Invitrogen) according to the manufacturer’s instructions. After treatment with gDNA wiper (Vazyme Biotech) to remove genomic DNA, RNA was transcribed into cDNA using the HiScriptIII qRT SuperMix kit (Vazyme Biotech). For semiquantitative real-time PCR detection of gene expression, cDNA samples were mixed with primer pairs and ChamQ Universal SYBR qPCR Master Mix (Vazyme Biotech). PCR amplification was conducted using Roche LightCycler 96 instruments. The following primers were used: *Mafb* sense (5’-

ACG AGG TGA TCC GCC TGA AG-3') and *Mafb* anti-sense (5'- GAG CTG CGT CTT CTC GTT CT -3'); *Maf* sense (5'- GAC TAC TAC TGG ATG ACC GG -3') and *Maf* anti-sense (5'- ATA GCC ATC GAA GCC ACC CT -3'); *Tnfa* sense (5'- ATG AGC ACA GAA AGC ATG ATC -3') and *Tnfa* anti-sense (5'- TAC AGG CTT GTC ACT CGA ATT -3'); *Il4* sense (5'- GAT GTG CCA AAC GTC CTC AC -3') and *Il4* anti-sense (5'- CTT GGA AGC CCT ACA GAC GA -3'); *Csf1* sense (5'- CGC GGC CGG AAA GTG A -3') and *Csf1* anti-sense (5'- GCC CAG CCA TGT CGA AGA AG -3'); *Csf2* sense (5'- GAC AGC GGA AGA CAA ACG AG -3') and *Csf2* anti-sense (5'- TCC CAA TAG CTC CCC CAC TC -3'); *Il6* sense (5'- GAT GCT ACC AAA CTG GAT ATA ATC -3') and *Il6* anti-sense (5'- GGT CCT TAG CCA CTC CTT CTG TG -3'); *Ccl2/Mcp1* sense (5'- GGG CCT GTT GTT CAC AGT TG -3') and *Ccl2/Mcp1* anti-sense (5'- GGG CAT TAA CTG CAT CTG GC -3'); *Inos* sense (5'- TGC CAG GGT CAC AAC TTT ACA -3') and *Inos* anti-sense (5'- CAG CTC AGT CCC TTC ACC AA -3'); *Arg1* sense (5'- CCT GCG ACC CAA GAA GAC TA -3') and *Arg1* anti-sense (5'- TTT GAG AAA GGC GCT CCG AT -3'); *Fizz1* sense (5'- GAC TGC TAC TGG GTG TGC TT -3') and *Fizz1* anti-sense (5'- GCT GGG TTC TCC ACC TCT TC -3'); *Mrc2* sense (5'- ACA GCT TCT TGC CAT CAG CA -3') and *Mrc2* anti-sense (5'- CAC GCC CTT GGC TCT ATT CT -3'); *Il1b* sense (5'- TGC CAC CTT TTG ACA GTG ATG -3') and *Il1b* anti-sense (5'- AAG GTC CAC GGG AAA GAC AC -3'); *Cxcl1* sense (5'- TCC AGA GCT TGA AGG TGT TGC C -3') and *Cxcl1* anti-sense (5'- AAC CAA GGG AGC TTC AGG GTC A -3'); *Cxcl2* sense (5'- GCT GTC CCT CAA CGG AAG AA -3') and *Cxcl2* anti-sense (5'- CAG GTA CGA TCC AGG CTT CC -3'); *Gapdh* sense (5'- CAA CTT TGG CAT TGT GGA AGG -3') and *Gapdh* anti-

sense (5’- GAT GCA GGG ATG ATG TTC TGG -3’); Human *KI67* sense (5’- CGT CCC AGT GGA AGA GTT GT -3’) and Human *KI67* anti-sense (5’- CGA CCC CGC TCC TTT TGA TA -3’); Human *CD80* sense (5’- AAA CTC GCA TCT ACT GGC AAA -3’) and Human *CD80* anti-sense (5’- GGT TCT TGT ACT CGG GCC ATA-3’); Human *IL1B* sense (5’- TTC GAC ACA TGG GAT AAC GAG G -3’) and Human *IL1B* anti-sense (5’- TTT TTG CTG TGA GTC CCG GAG -3’); Human *IL6* sense (5’- ATG TCT GAG GCT CAT TCT GCC -3’) and Human *IL6* anti-sense (5’- TGG AAT CTT CTC CTG GGG GT-3’); Human *MAF* Human sense (5’-CTG GCA ATG AGC AAC TCC GA-3’) and Human *MAF* Human anti-sense (5’-AGC CGG TCA TCC AGT AGT AGT-3’); Human *MAFB* Human sense (5’-TCA AGT TCG ACG TGA AGA AGG-3’) and Human *MAFB* Human anti-sense (5’-GTT CAT CTG CTG GTA GTT GCT-3’); *GAPDH* sense (5’- GTC AAG GCT GAG AAC GGG AA -3’) and Human *GAPDH* anti-sense (5’- AAA TGA GCC CCA GCC TTC TC -3’).

ALT/AST test. Blood samples were collected via cardiac puncture or from the orbital venous plexus and were stored at 4°C overnight. Serum was then obtained from blood by centrifugation at 3000 × g for 5 minutes. ALT/AST levels were measured using commercially available diagnostic kits (Nanjing Jiancheng Bio. Co.) according to the manufacturer’s instructions.

Movies 1 to 5

Movie 1: KCs rapidly captured *E. coli* regardless of the size of the bacterial inoculum.

Movie 2: KCs efficiently captured LPS-attenuated *E. coli* bacteria.

Movie 3: Metastatic tumor cells were arrested and ripped off by KCs.

Movie 4: KCs intimately interacted with and nibbled tumor cells upon bacterial therapy.

Movie 5: DKO macrophages nibbled tumor cells during co-culture

Supplemental Figures and Legends 1-16

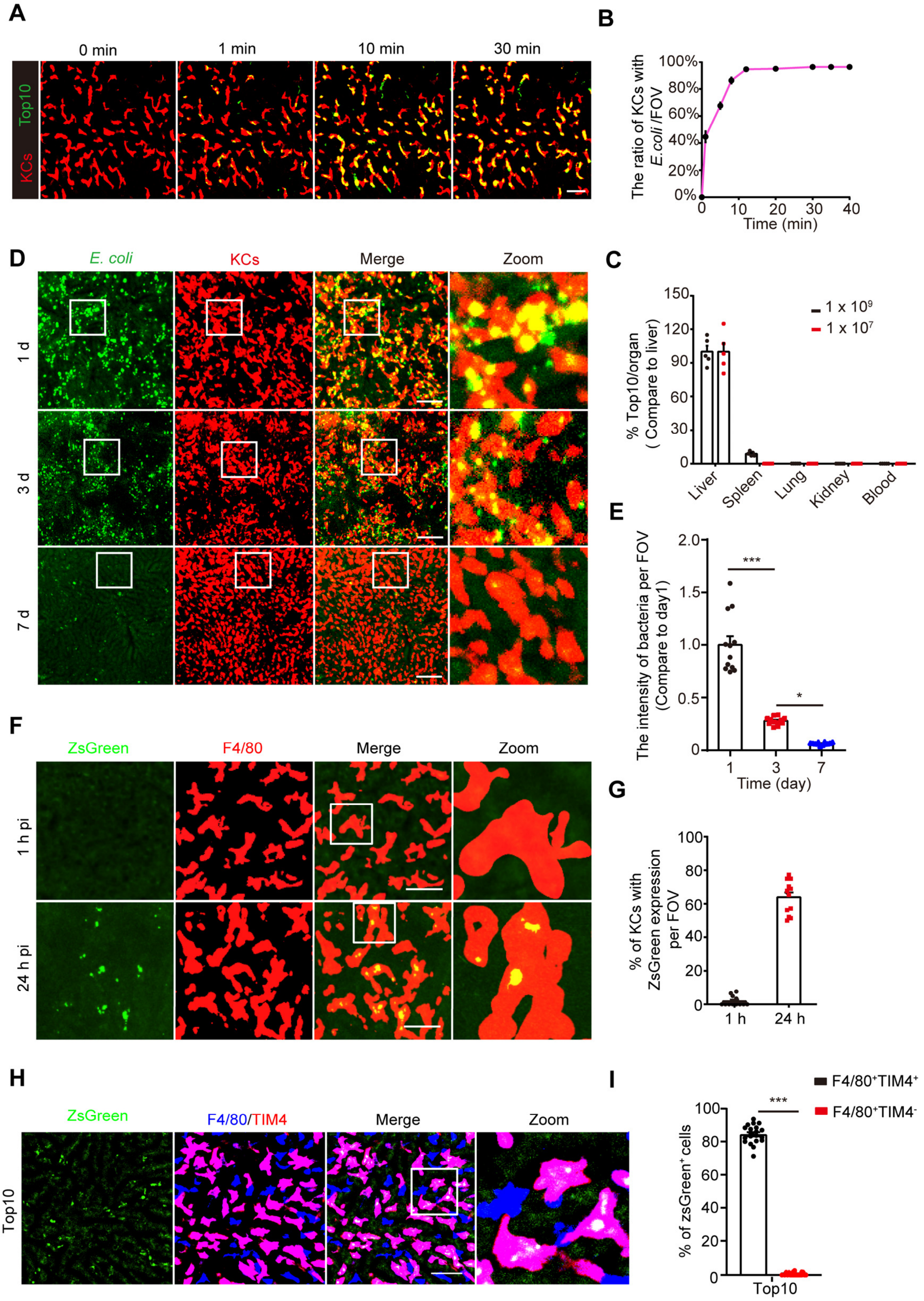

Supplemental Figure 1. KC were able to capture and clear bacteria at a high infection dose.

(**A**) Time lapse liver images showing the capture of sfGFP-tagged *E. coli* by KCs at an

infection dose of 1×10^9 CFU; scale bars, 50 μm. (**B**) Quantification of the percentage of KCs that engulfed bacteria per FOV. (**C**) Mice were infected with 1×10^7 or 1×10^9 CFU *E. coli* Top10. Bacterial burden in organs at 1 h p.i. were measured and normalized to the bacterial CFU in the liver. n=5 mice per group. (**D**) Mice were infected with 1×10^9 CFU *E. coli* Top10, and representative intravital liver images were shown at day 1, day 3 or day 7 p.i.; scale bars, 50 μm. (**E**) The amounts of *E. coli* per FOV in (**D**) were quantified based on the normalized GFP fluorescence intensity. (**F**) Mice were infected with 1×10^9 CFU of *E. coli* Top10-harboring mammalian expression plasmids for ZsGreen Representative intravital liver images at 1 h and 24 h p.i. were shown; scale bars, 50 μm. (**G**) The percentage of KCs with ZsGreen expression was quantified. (**H**) Co-staining of F4/80 and TIM4 at 24 h post injection of *E. coli* Top10-harboring mammalian expression plasmids for ZsGreen; scale bars, 50 μm. (**I**) The percentage of $TIM4^+F4/80^+$ KCs with ZsGreen expression was quantified. For E, G and I, n=12 randomly selected FOVs from 3 mice per group. Representative results from at least two independent experiments were shown. Data were expressed as the mean ± SEM. **P* < 0.05, ****P* < 0.001, by One-way ANOVA with Tukey's test in **(E)**, and unpaired two-tailed Student's *t* test in **(I)**.

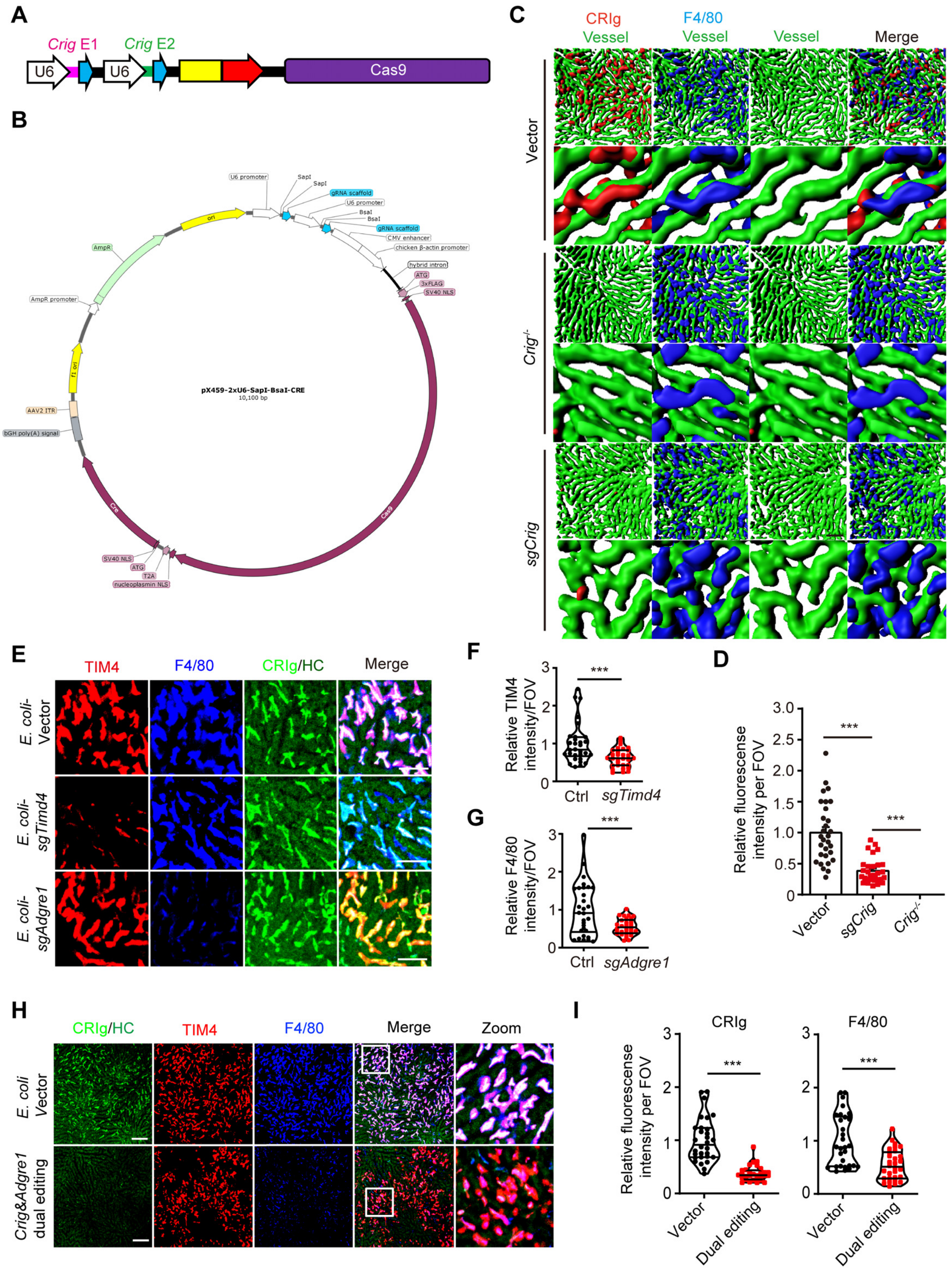

Supplemental Figure 2. In situ gene editing of KCs via bacterial-mediated delivery of CRISPR/Cas expressing plasmids.

(**A** and **B**) Schematic depiction of the constructed dual sgRNA CRISPR plasmid: pX459-2U6-SapI-BsaI-Cre. (**C**) 3D-images showing CRIg expression in control mice,

Crig edited mice and *Crig*$^{-/-}$ mice. Vessels were illuminated with FITC-dextran. Scale bars, 50 μm. (**D**) Normalized fluorescence intensity of CRIg and F4/80 per FOV, n=30 randomly selected FOVs. (**E**) Liver images at day7 post injection with 1×10^{9} CFU of Top10 harboring control, TIM4- or F4/80-targeting CRISPR plasmids. Scale bars, 60 μm. (**F** and **G**) Normalized fluorescence intensity of TIM4 or F4/80 per FOV, n=30 randomly selected FOVs. (**H**) Simultaneous editing of CRIg and F4/80 on KCs by injecting mice with *E. coli* Top10 harboring both pX459-2U6-2*sgCrig* and pX459-2U6-2*sgAdgre1* plasmids. Representative liver images at day 7 p.i. were shown; scale bars, 100 μm. (**I**) Normalized fluorescence intensity of CRIg and F4/80 per FOV, n=30 randomly selected FOVs. Representative results from at least two experiments were shown. Data were expressed as the mean ± SEM. ***P< 0.001, by unpaired two-tailed Student's *t* test in (**F**), (**G**) and (**I**), and one-way ANOVA with Tukey's test in (**D**).

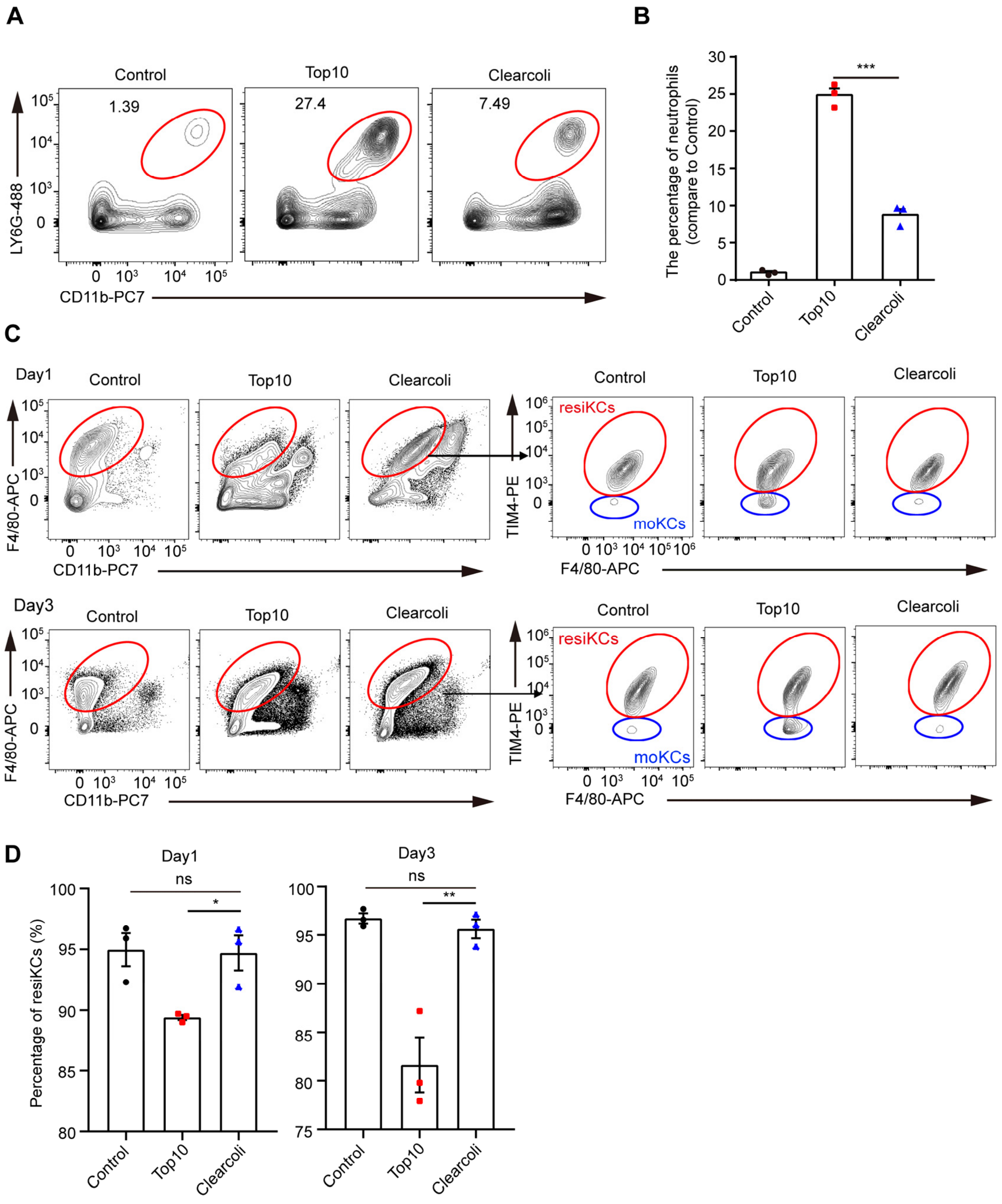

Supplemental Figure 3. ***E. coli*** **injection-induced endotoxemia was abolished by using Clearcoli.**

(**A** and **B**). Representative flow cytometric plots and statistics of hepatic neutrophils at 24 h after infection with 1×10^9 CFU of TOP 10 or Clearcoli. n=3 mice per group. (**C** and **D**). Representative flow cytometric plots and statistics of liver resident KCs ($CD45^+$ $Ly6G^-$ $CD11b^{low}$ $F4/80^+$ $TIM4^+$) at 24 h and 72 h after infection with Top10 or Clearcoli.

n=3 mice per group. Uninfected mice were used as controls. Data were expressed as the mean ± SEM; ns, no significant difference; *$P < 0.05$; **$P < 0.01$; ***$P < 0.001$, by one-way ANOVA with Tukey's test.

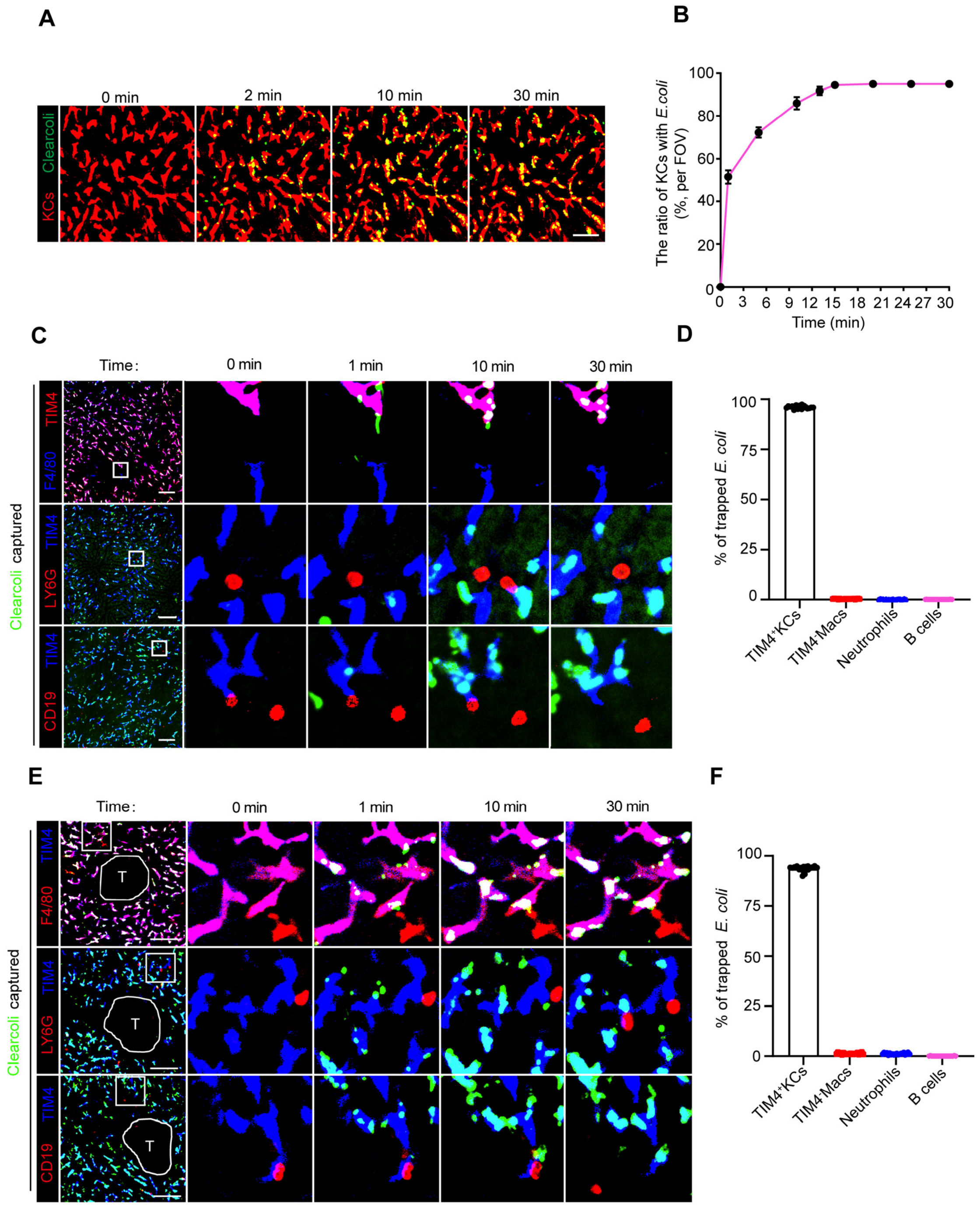

Supplemental Figure 4. Clearcoli were captured exclusively by resident KCs.

(**A**) Time-lapse liver images showing the capture of sfGFP-tagged Clearcoli by KCs at an infection dose of 1×10^9 CFU; scale bars, 50 μm. (**B**) Quantification of the percentage of KCs that engulfed Clearcoli per FOV at the indicated time points. (**C** and **D**) Representative intravital images and statistics of Clearcoli caught by TIM4$^+$ resident

KCs, TIM4$^-$macrophages, neutrophils or B cells in tumor-free WT mice; scale bars, 100 μm, n=30 randomly selected FOVs. (**E** and **F**) Mice were intrasplenically inoculated with B16F10 tumors for 7 days and then subjected to high-dose Clearcoli injection. Representative intravital images and statistics of bacterial capture by TIM4$^+$ resident KCs, TIM4$^-$macrophages, neutrophils or B cells were shown; “T” indicates the tumor loci. Scale bars, 100 μm, n = 20 FOVs per group. Data were expressed as the mean ± SEM.

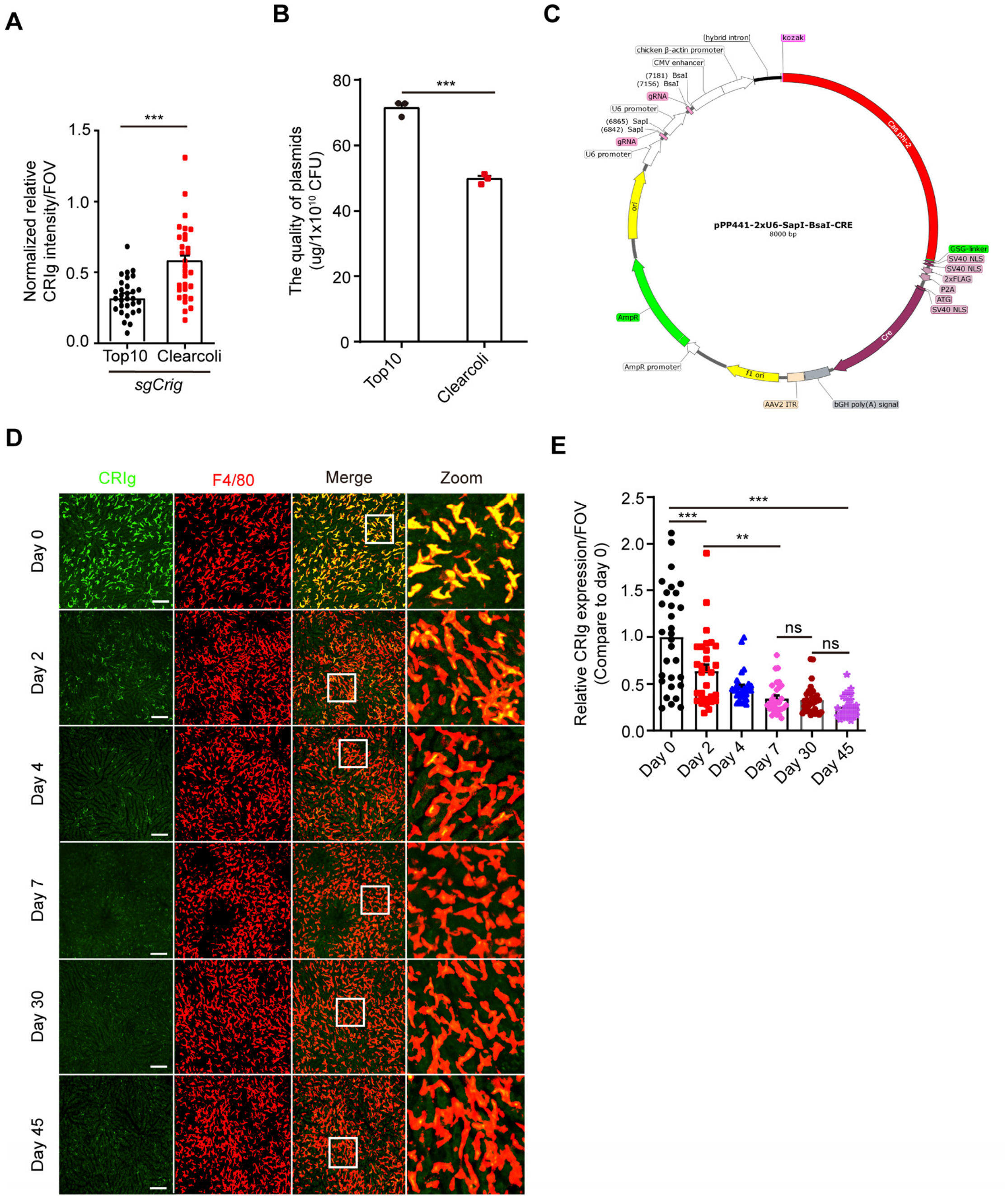

Supplemental Figure 5. The Clearcoli-CRISPR/CasΦ system induced highly efficient and long-lasting gene editing in KCs

(**A**) Mice were injected with 1×10^9 CFU of Top10-pX459-2U6, Top10-pX459-2U6-2*sgCrig*, Clearcoli-pX459-2U6 or Clearcoli-pX459-2U6-2*sgCrig* bacteria. Hepatic expression of CRIg in these mice was detected using IVM at 7 days p.i., and were presented as the fluorescence intensity normalized to vector controls. Each dot

represented one FOV, a total of 30 FOVs from 3 mice per group were analyzed. (**B**) Top10-or Clearcoli transformed with pX459-2U6-2*sgCrig* plasmids were cultured overnight. Plasmids extracted from 1×10^{10} CFU of each bacterial strain were measured. Each dot represented one bacterial sample. (**C**) Schematic depiction of the constructed dual sgRNA CRISPR plasmids: pPP441-2U6-SapI-BsaI-Cre. (**D**) Mice were injected with 1×10^{9} CFU of Clearcoli harboring pPP441-2U6-2*sgCrig*, CRIg expression in the liver was detected at day 0, 2, 4, 7, 30 and 45; scale bars, 100 μm. (**E**) Normalized fluorescence intensity of CRIg per FOV. A total of 30 FOVs from 3 mice per group were analyzed. Data were expressed as the mean ± SEM; ns, no significant difference; *$P < 0.05$; **$P < 0.01$; ***$P < 0.001$, by unpaired two-tailed Student's *t* test in (**A**) and (**B**), and one-way ANOVA with Tukey's test in (**E**).

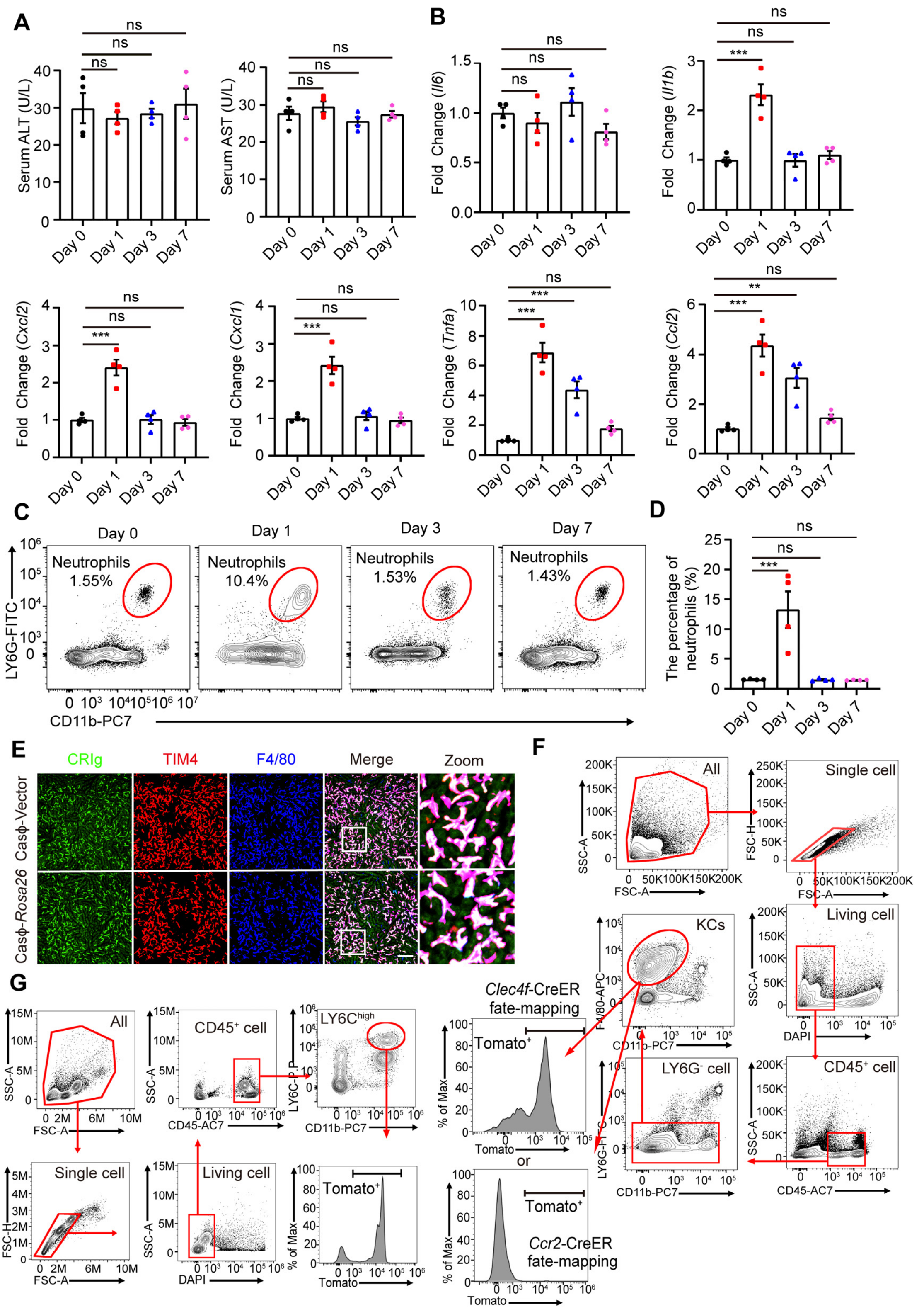

Supplemental Figure 6. Clearcoli-CRISPR/CasΦ system-mediated gene editing in KCs did not induce robust inflammation.

Mice were injected with 1×10^9 CFU of Clearcoli transformed with pPP441-2U6-

2*sgRosa26* plasmids for the purpose of gene editing in KCs; (**A**) Serum levels of ALT and AST; (**B**) hepatic mRNA levels of *Il1b*, *Cxcl1*, *Cxcl2*, *Ccl2*, *Tnfa* and *Il6*; and (**C** and **D**) intrahepatic recruitment of neutrophils were detected at day 0, 1, 3 and 7, n=4 mice per group. (**E**) Hepatic expression of CRIg, TIM4 and F4/80 at day 7 post-injection with Clearcoli harboring either the control CRISPR/CasΦ or *Rosa26*-targeting CRISPR/CasΦ plasmids; scale bars, 100 μm. (**F**) Gating strategy and labeling efficiency for KCs in *Clec4f*-creER:RCL-tdT mice and *Ccr2*-creER:RCL-tdT mice after TAM induction, related to Figure 2J-K. (**G**) Efficient labeling of blood monocytes in *Ccr2*-creER:RCL-tdT mice after TAM induction, related to Figure 2J. Data were expressed as the mean ± SEM. ns, no significant difference, ***P* < 0.01, ****P* < 0.001, by one-way ANOVA with Tukey's test.

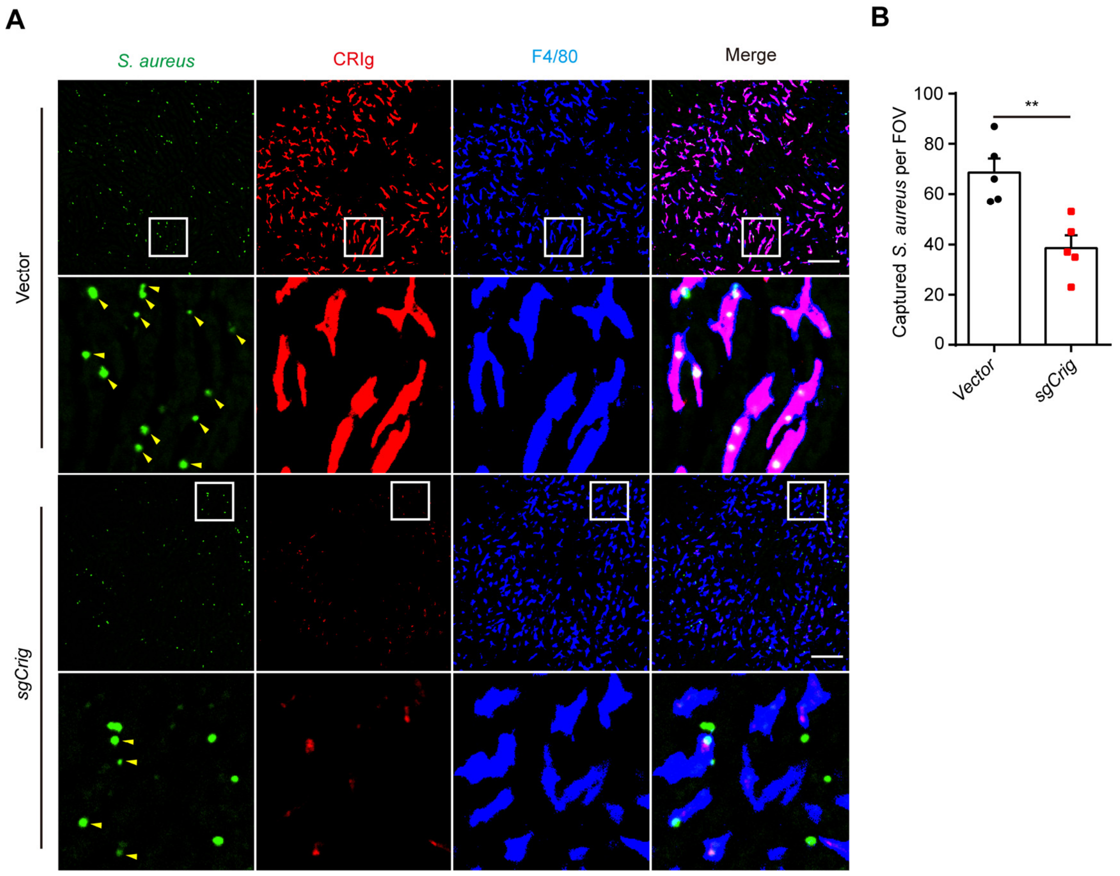

Supplemental Figure 7. Impaired capture of *S. aureus* by KCs upon BIL-CRISPR mediated disruption of CRIg expression.

(**A**) Mice were treated with Clearcoli-vector or Clearcoli-*sgCrig* for 7 days allowing for gene editing of CRIg in KCs, followed by infection with 5×10^7 CFU of GFP-tagged *S. aureus* MW2. Representative intravital liver images and enlarged pictures at 10 minutes post infection were shown. Yellow arrow indicated *S. aureus* captured by KCs; scale bars, 100 μm. (**B**) Quantification of the number of *S. aureus* captured by KCs per FOV. n=5 mice per group were shown. Data were expressed as the mean ± SEM; $^{**}P < 0.01$, by unpaired two-tailed Student's *t* test.

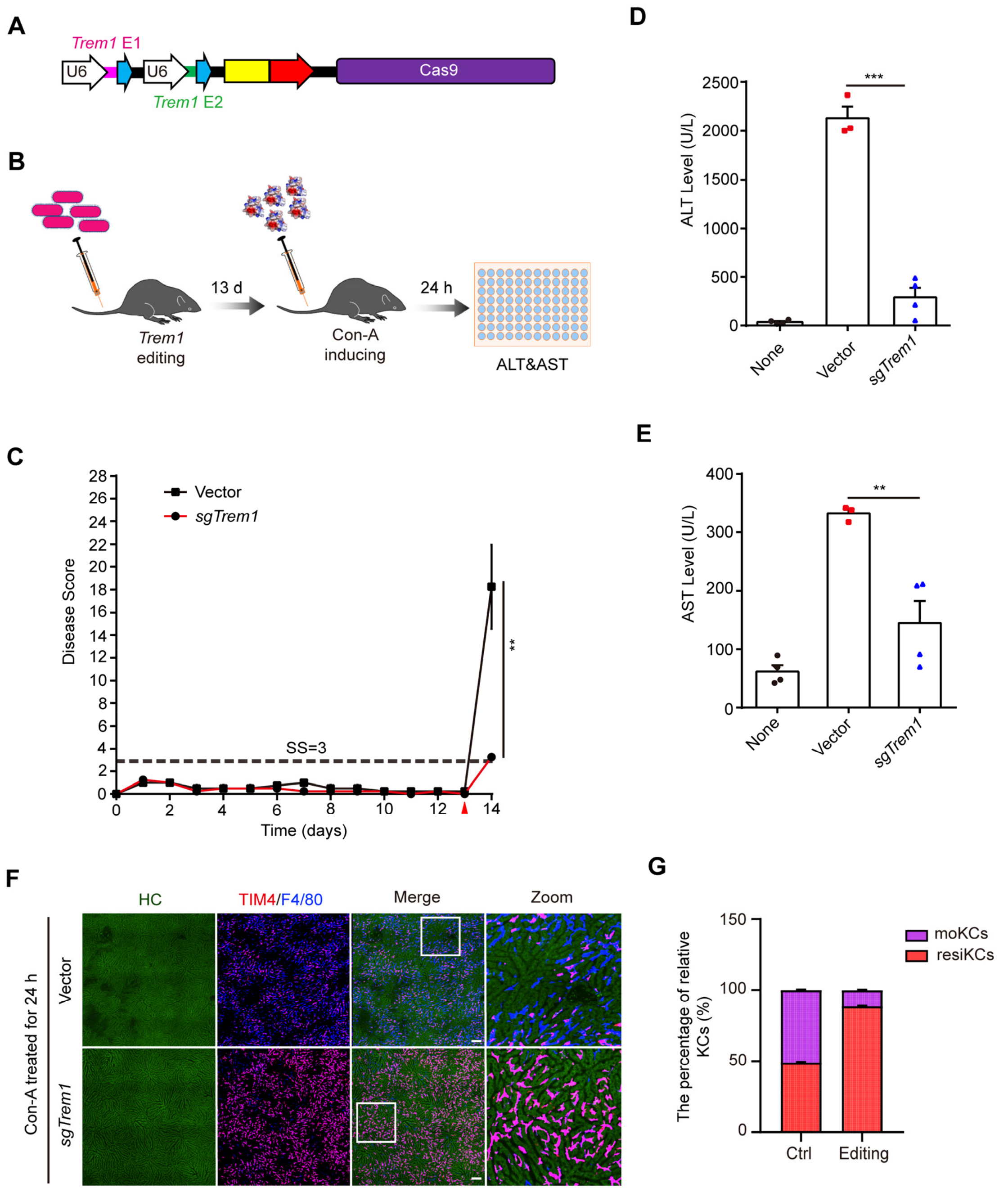

Supplemental Figure 8. BIL-CRISPR-mediated disruption of Trem1 in KCs prevented Con-A-induced hepatitis.

(**A**) Schematic illustration of the *Trem1*-targeting dual sgRNA CRISPR vectors. (**B**) Design of the experiment. Mice were treated with *E. coli* containing either vector or *Trem1*-editing plasmids for 13 days, followed by Con-A injection. (**C**) The disease score of mice was monitored. The red arrow indicated the timepoint of Con-A injection. Mice

that scored less than 3 were considered as healthy, and mice that scored more than 18 was euthanized. n=4 mice per group were shown. (**D** and **E)** Serum levels of ALT and AST were measured at 24 h post-Con-A injection. n=3 mice in the vector group (one mouse was euthanized before harvesting), n=4 mice in *Trem1*-editing group; mice without any treatment were used as negative controls (n=4). (**F**) Representative intravital images showing the distribution of KC in control or *Trem1*-editing livers at 24h post-Con-A injection; scale bars, 100 μm. (**G**) The percentage of F4/80$^+$TIM4$^+$ resident KCs and F4/80$^+$TIM4$^-$ macrophages in F. Data were expressed as the mean ± SEM; $^{**}P < 0.01$, $^{***}P < 0.001$, by Student's *t* test in (**C**), and one-way ANOVA with Tukey's test in (**D**) and (**E**).

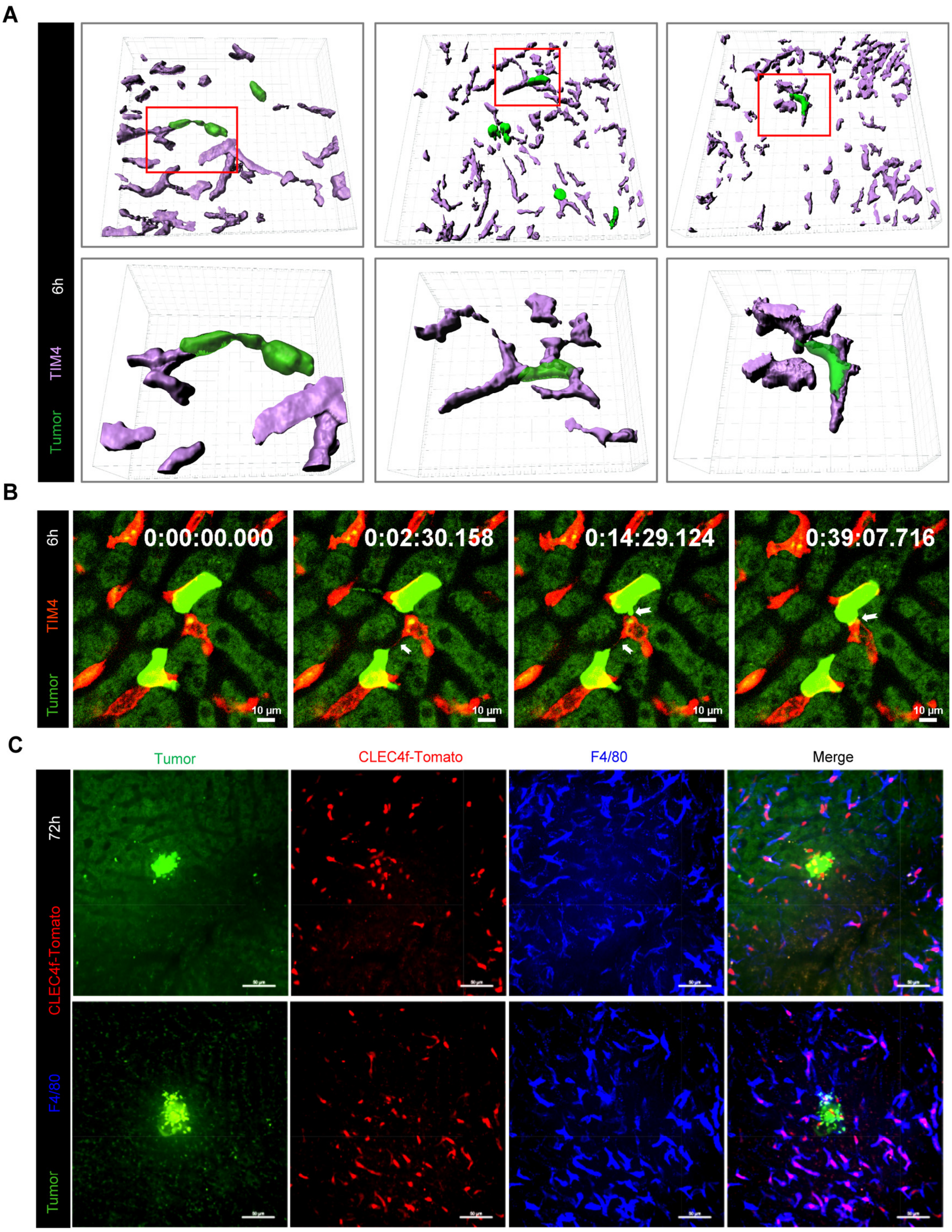

Supplemental Figure 9. KCs were pivotal for the early control of liver metastasis by arresting and ingesting cancer cells.

(**A**) Representative 3D intravital images of KC and tumor cell interactions at 6 h after intrasplenic injection of 3×10^5 B16F10-ZsGreen tumor cells. Enlarged images showing

that tumor cells were arrested but not fully engulfed by KCs at this time point. (**B**) Time lapse intravital images of the interactions between tumor cells and KCs in A. White arrows indicate that arrested tumor cells ripped off by neighboring KCs. Scale bars, 10 μm. (**C**) *Clec4f*-tdTomato mice were intrasplenically injected with 3×10^5 B16F10-ZsGreen tumor cells, and IVM of mouse liver was performed 72 hours later. The upper image shows intratumoral infiltration of KCs, and the lower image shows that a small metastatic tumor encased by KCs undergoes dissociation. Scale bars, 50 μm.

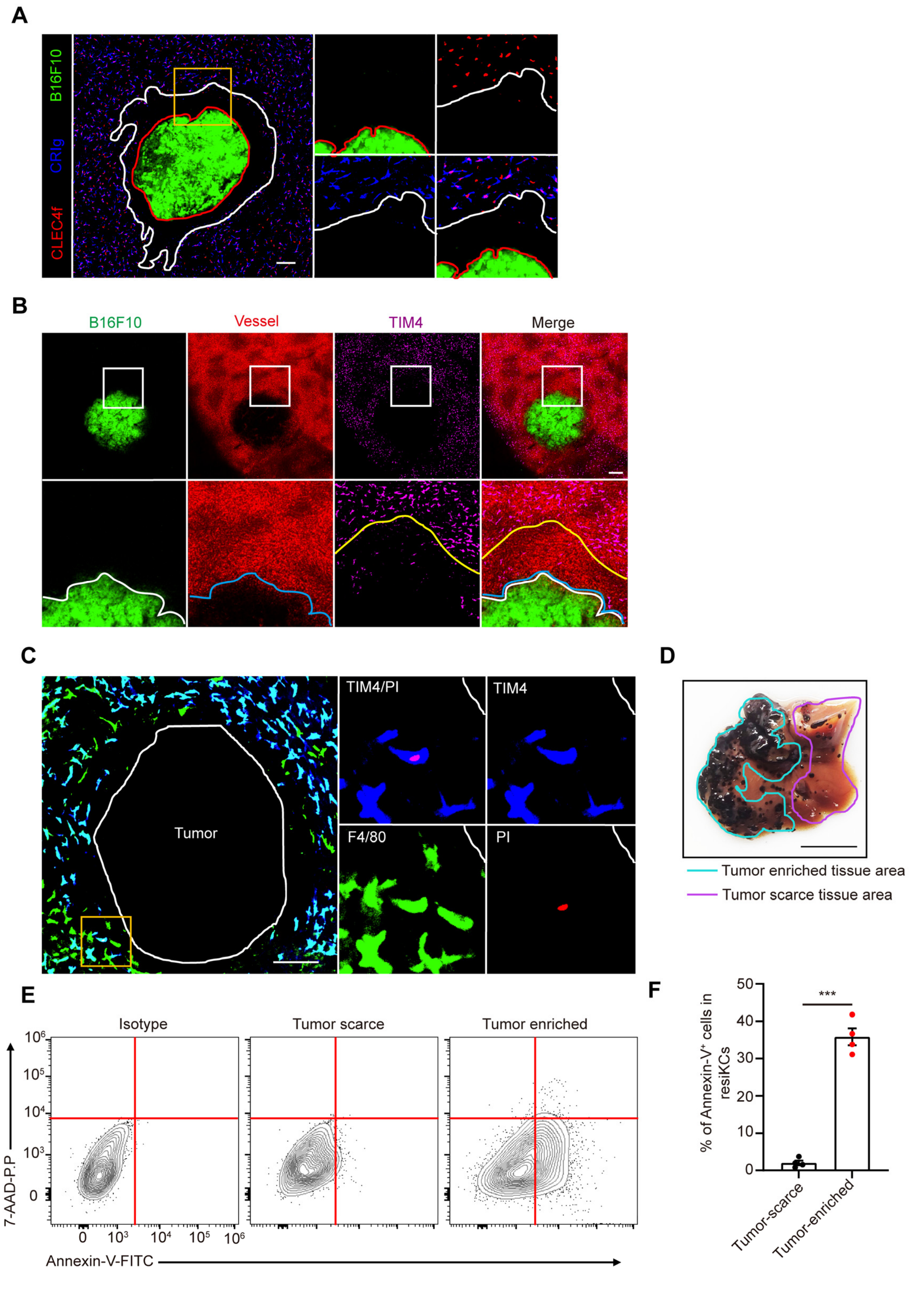

Supplemental Figure 10. Formation of the KC "dark zone" in the periphery of macrometastasis.

(**A**) *Clec4f*-tdTomato mice were labeled with Af647-conjugated CRIg to show the

existence of KC dark zones in the tumor periphery; scale bar, 100 μm. (**B**) B16F10-ZsGreen tumor-bearing mice were injected *i.v.* with 50 mg/kg body weight TRITC-dextran for one hour, and intravital liver images are shown; scale bar, 200 μm. (**C**) B16F10-ZsGreen tumor-bearing mice were stained with PI (1.25 mg/kg *i.v*) for one hour; scale bar, 100 μm. (**D**) Tumor-enriched and tumor-scarce liver tissues in B16F10-bearing mice were harvested, and (**E** and **F**) flow cytometry was performed to measure Annexin-V and 7-AAD expression in KCs from these tissues, n=4 mice per group. Scale bar, 1 cm. Data were expressed as the mean ± SEM; ***$P < 0.001$, by Student's *t* test.

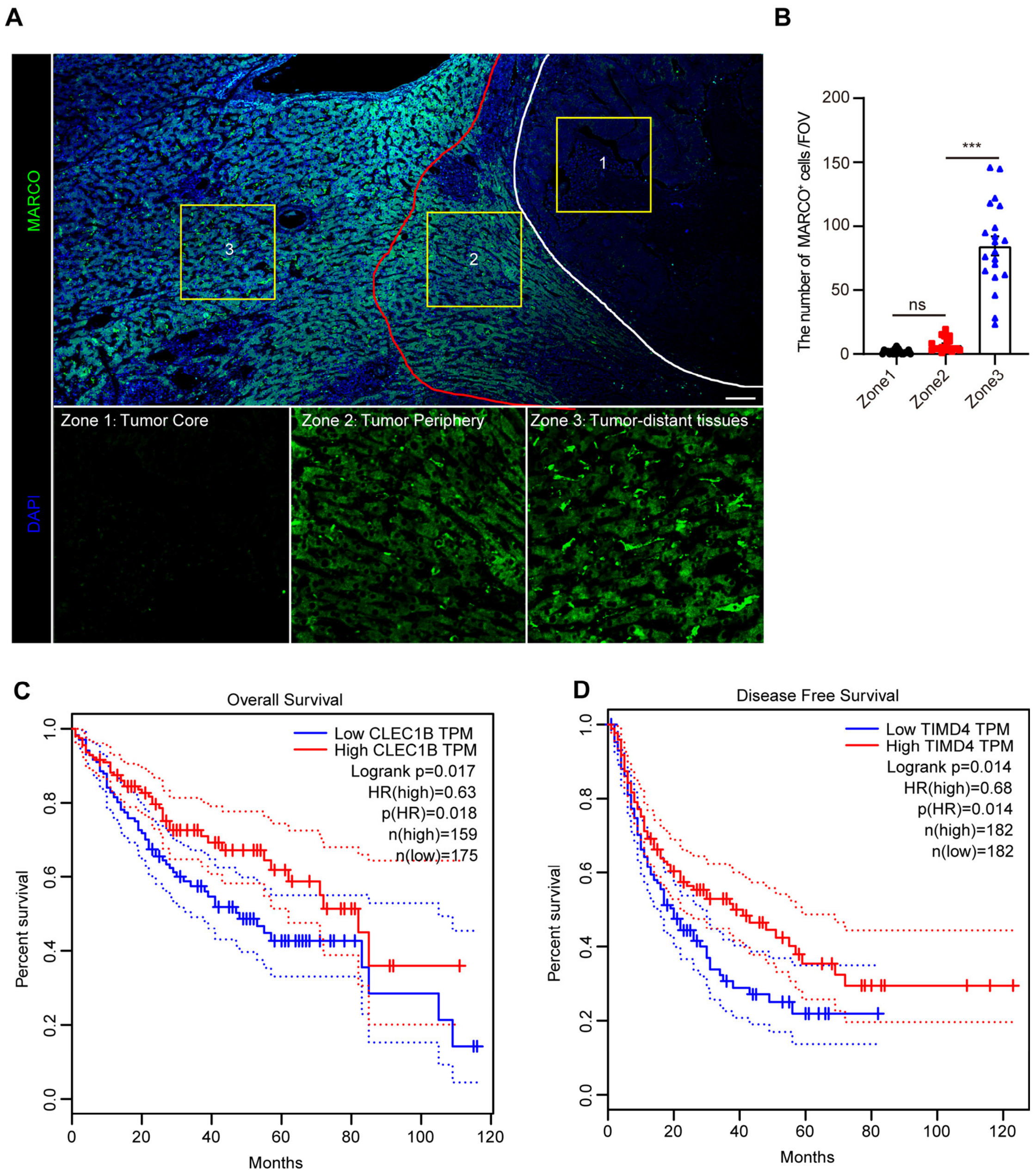

Supplemental Figure 11. Decreased KC abundance in the tumor core and periphery of liver sections from patients with CRC liver metastasis.

(**A**) Representative images of MARCO staining of liver sections from patients with CRC liver metastasis. Zone 1, 2 and 3 represents the tumor core, tumor periphery and tumor distant liver tissues respectively; scale bars, 100 μm. (**B**) The number of KCs in different zones of liver sections were calculated, n=20 FOV from 6 patient-derived

sections. (**C**) Overall survival of LIHC patients with high *CLEC1B* expression (cutoff-high [%] = 50) and low *CLEC1B* expression (cutoff-low [%] = 50); (**D**) Disease-free survival of LIHC patients with high *TIMD4* expression (cutoff-high [%] = 50) and low *TIMD4* expression (cutoff-low [%] = 50). Data were expressed as the mean ± SEM; ns, no significant difference, ***$P < 0.001$, by One-way ANOVA with Tukey's test.

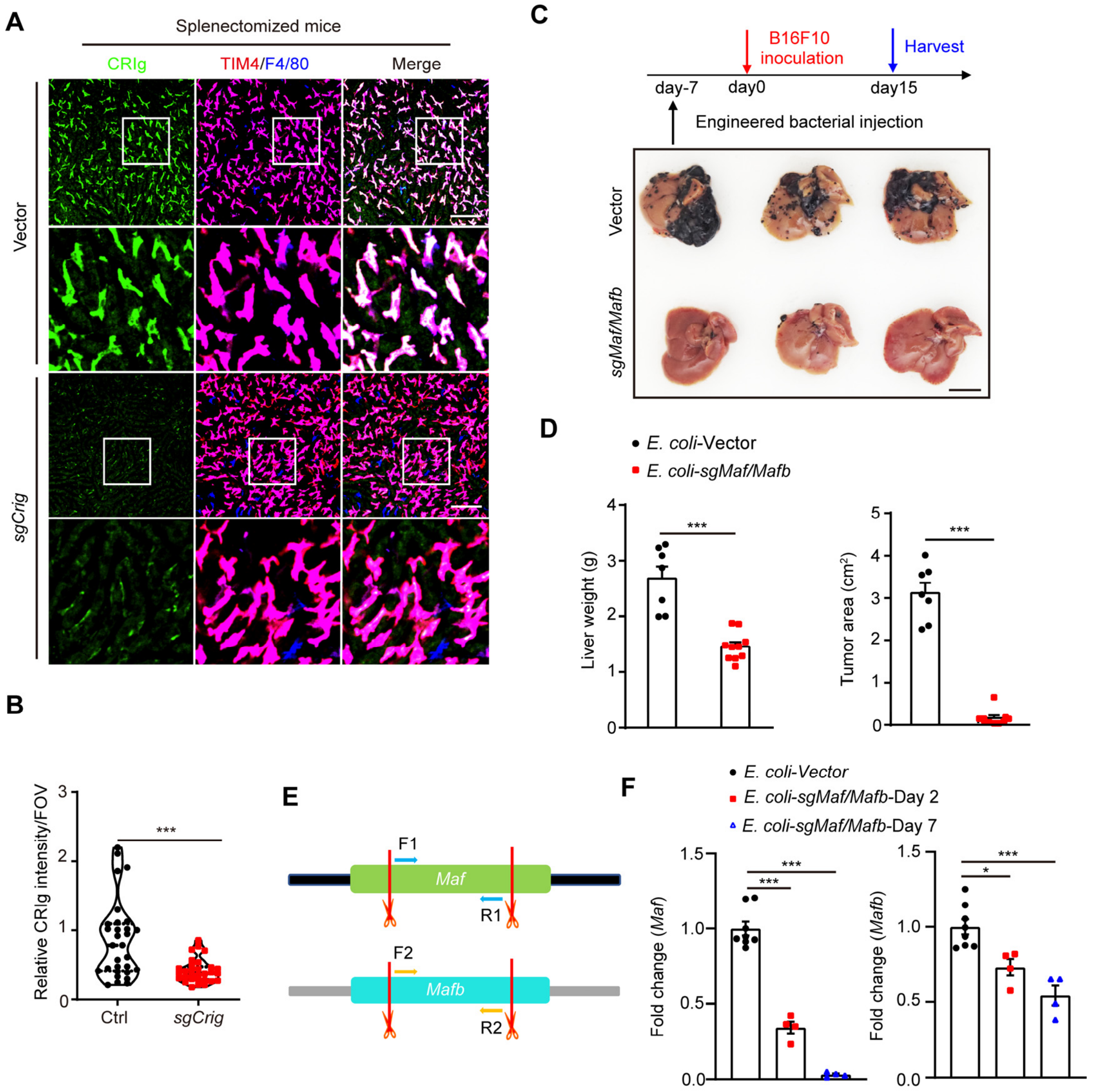

Supplemental Figure 12. *Maf/Mafb* deletion in KCs using BIL-CRISPR conferred full protection against liver metastasis in mice.

(**A**) Representative IVM images showing CRIg expression in splenectomized mice after injection of Clearcoli harboring *Crig*-editing plasmids or control vectors; scale bars, 100 μm. (**B**) Relative intensity of CRIg per FOV in A. n=30 FOV from 3 mice. (**C**) Mice were injected with 1×10^9 CFU of Clearcoli harboring either backbone vector or *Maf/Mafb* editing plasmids for 7 days, followed by intrasplenic injection of 3×10^5 B16F10 cells. The livers were harvested at day 15 as depicted; scale bar, 1 cm. (**D**)

Liver weights and the tumor areas on the surface of the liver were measured. n=7-10 mice per group pooled from two experiments were shown. (**E**) Schematic depiction of the design of primer pairs for real-time PCR detection of *Mafb* and *Maf* deletion in KCs. (**F**) Tissue-resident KCs from *E. coli*-vector- or *E. coli*-*sgMaf/Mafb*-treated tumor bearing mice were sorted, and the normalized mRNA expression of *Maf* and *Mafb* at day 2 and day 7 after bacterial treatment was shown; n=8 mice in the vector group, and n=4 mice in the day 2 or day 4 group. Data were expressed as the mean ± SEM; *$P < 0.05$, ***$P < 0.001$, by Unpaired two-tailed Student's *t* test in (**B**) and (**D**), One-way ANOVA with Tukey's test in (**F**).

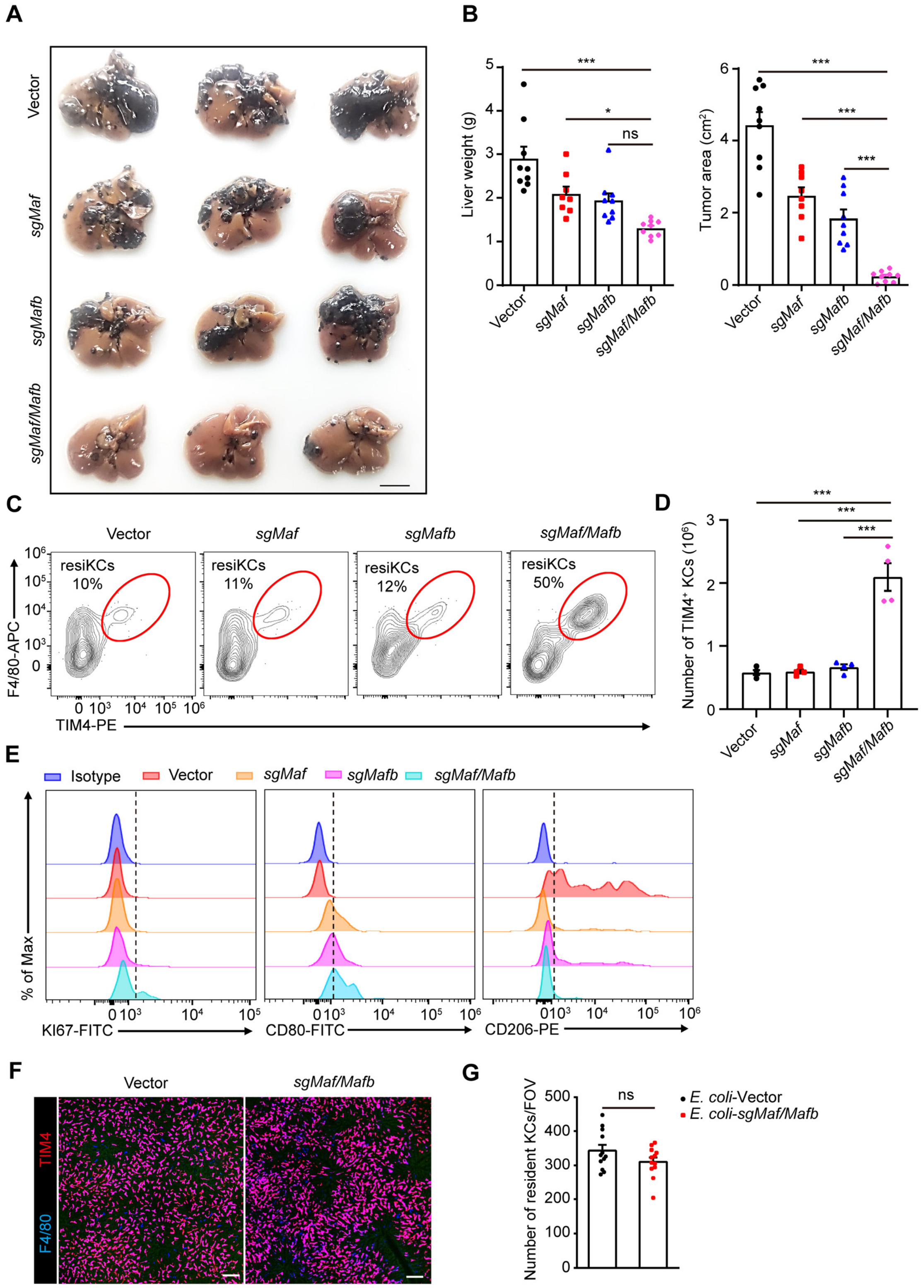

Supplemental Figure 13. *Maf* or *Mafb* editing alone exhibited suboptimal therapeutic effects against liver metastasis.

(**A**) Mice with established B16F10 liver metastasis were treated with *E. coli*-vector, *E.*

coli-sgMaf, *E. coli-sgMafb* or *E. coli-sgMaf/Mafb* at day 12 post tumor inoculation and harvested at day 19; scale bar, 1 cm. (**B**) Liver weights and the tumor areas were measured; n=8-9 mice per group pooled from two independent experiments. (**C**) Representative flow cytometric plots of KCs in A, gated from DAPI$^-$CD45$^+$Ly6G$^-$ singlets. (**D**) The number of TIM4$^+$F4/80$^+$ tissue resident KCs and their expression of **(E)** KI67, CD80 and CD206 were shown; n=4 mice per group. (**F**) Tumor-free C57BL/6 mice were injected with 1×10^9 CFU of Clearcoli harboring either backbone vector or *Maf/Mafb* editing CRISPR plasmids for 7 days. Representative intravital liver images are shown; scale bars, 120 μm. (**G**) Quantification of the number of TIM4$^+$ KCs per FOV in F; a total of 12 randomly selected FOVs from 3 mice per group were analyzed. Data are expressed as the mean ± SEM; ns, no significance, *$P < 0.05$, ***$P < 0.001$, by One-way ANOVA with Tukey's test in (**B**) and (**D**), and unpaired two-tailed Student's *t* test in (**G**).

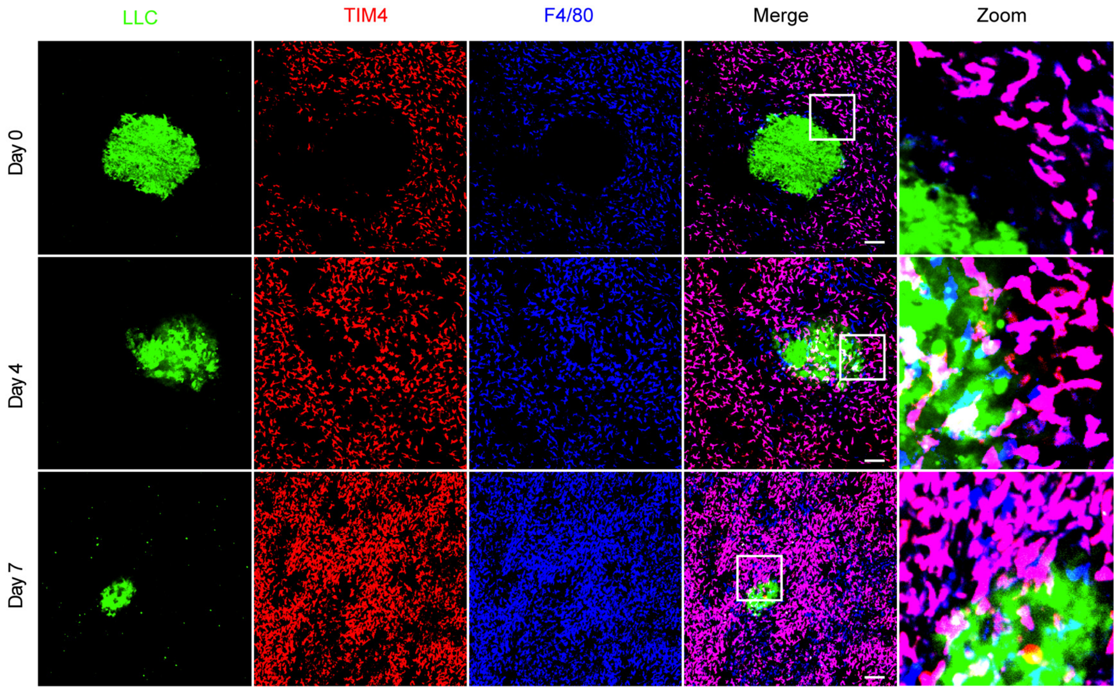

Supplemental Figure 14. KC-dependent elimination of LLC liver metastasis after *Maf/Mafb* editing.

Mice with established LLC liver metastasis were treated with *E. coli*-vector or *E. coli*-*sgMaf/Mafb* at day 7 post tumor inoculation and intravital liver images of LLC-KC interfaces at various time points post bacterial treatment were shown; scale bars, 100 μm. Representative images from 3 mice per time points were shown.

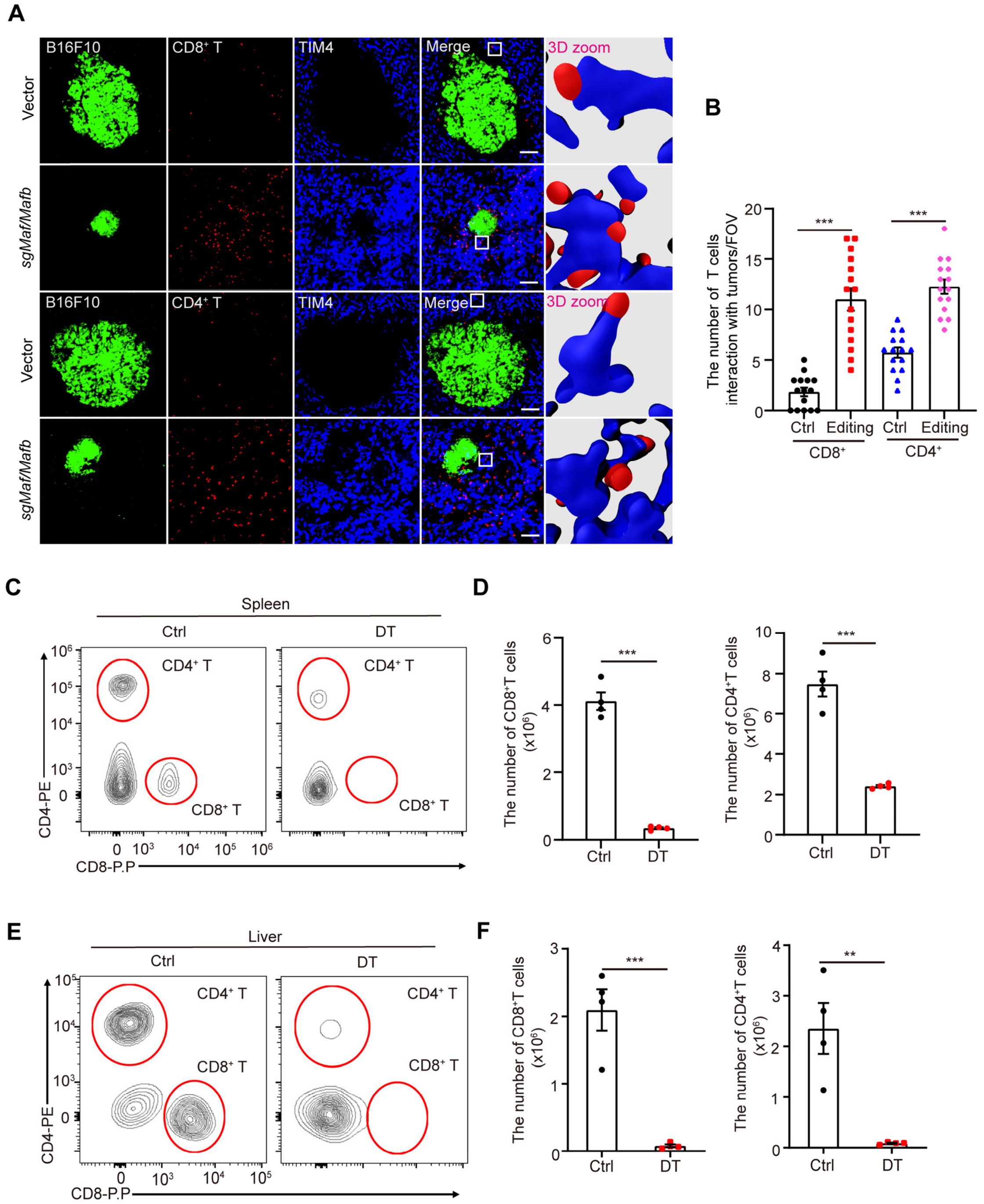

Supplemental Figure 15. Increased T cell infiltration during bacterial therapy.

(**A**) Mice with established B16F10 liver metastasis were treated with *E. coli*-vector or *E. coli-sgMaf/Mafb*. Representative intravital liver images of CD4⁺T cells, CD8⁺T cells, KCs and their interactions at 7 days post-bacterial treatment are shown; scale bars, 100 μm. (**B**) The number of T cells interacting with tumors per FOV was quantified. A

total of 15 randomly selected FOVs from 3 mice per group were analyzed. (**C-F**) Validation of T cell ablation in tumor bearing *Cd4*-Cre iDTR mice, related to Figure 7I-J. n=4 mice per group. Data are expressed as the mean ± SEM; $^{**}P < 0.01$, $^{***}P < 0.001$, by Two-way ANOVA with Tukey's test in (**B**), and Student's *t* test in (**D**) and (**F**).

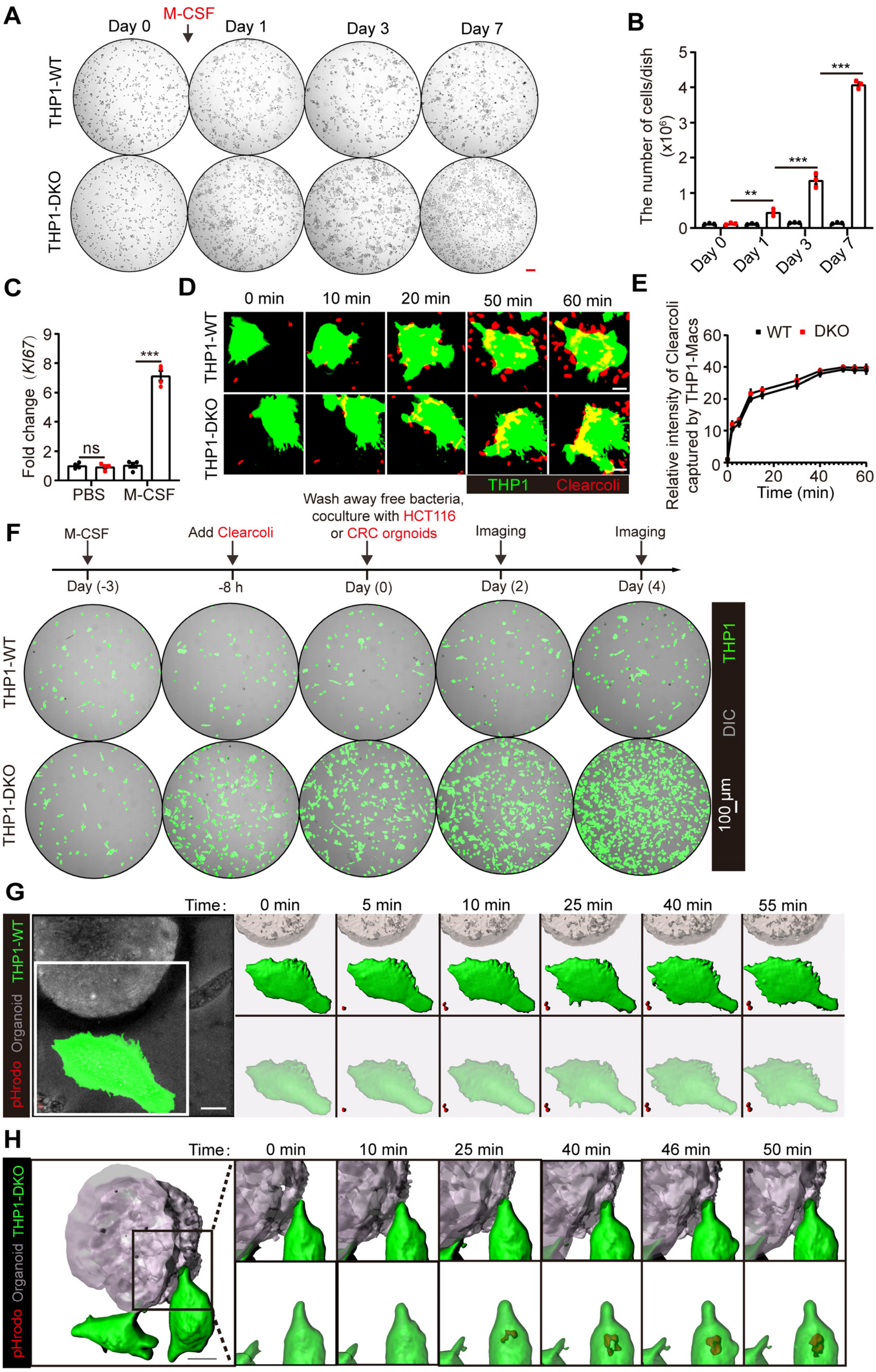
A
M-CSF
Day 0
Day 1
Day 3
Day 7
THP1-WT
THP1-DKO
B
The number of cells/dish (x10^6)
Day 0
Day 1
Day 3
Day 7
**
***
***
C
Fold change (Ki67)
ns
***
PBS
M-CSF
D
0 min
10 min
20 min
50 min
60 min
THP1-WT
THP1-DKO
THP1
Clearcoli
E
Relative intensity of Clearcoli captured by THP1-Macs
WT
DKO
Time (min)
F
M-CSF
Add Clearcoli
Wash away free bacteria, coculture with HCT116 or CRC orgnoids
Imaging
Imaging
Day (-3)
-8 h
Day (0)
Day (2)
Day (4)
THP1-WT
THP1-DKO
THP1
DIC
100 μm
G
Time:
0 min
5 min
10 min
25 min
40 min
55 min
pHrodo Organoid THP1-WT
H
Time:
0 min
10 min
25 min
40 min
46 min
50 min
pHrodo Organoid THP1-DKO

Supplemental Figure 16. Disruption of MafB AND c-Maf enhanced the anti-tumor activity of macrophages during M-CSF and Clearcoli treatment in vitro.

(**A**) Bright-field images of WT or DKO THP1 macrophages during human M-CSF treatment at the indicated time points. Scale bar, 100 μm. (**B**) Quantification of the number of macrophages in A. n=3 cell samples. (**C**) Q-PCR detection of *KI67* expression in WT or DKO THP1 macrophages 3 days after hM-CSF stimulation. n=4 cell samples. (**D**) Real-time imaging showing comparable capacity of WT or DKO THP1 macrophages to phagocytose Clearcoli *in vitro*; scale bars, 10 μm. (**E**) Relative fluorescence intensity of Clearcoli phagocytosed by WT or DKO THP1 macrophage, n=10 FOVs. (**F**) The experimental design for macrophage-tumor co-culture, related to Figure 8. WT or DKO THP1 macrophages were stimulated with hM-CSF for 3 days. Clearcoli were added during the last 8 h and washed away thoroughly. HCT116 or CRC organoids were then co-cultured with macrophages in the presence of hM-CSF. (**G** and **H)** 3D reconstruction of WT or DKO macrophages with pHrodo-prestained CRC organoids at 8 hours post co-culture; scale bar, 20 μm. Data are expressed as the mean ± SEM. ns, no significance, $^{**}P < 0.01$, $^{***}P < 0.001$, by Two-way ANOVA with Tukey's test in (**B**) and (**C**).
